# *Lonicerae Japonicae Flos* with the homology of medicine and food: a review of active ingredients, anticancer mechanisms, pharmacokinetics, quality control, toxicity and applications

**DOI:** 10.3389/fonc.2024.1446328

**Published:** 2024-09-09

**Authors:** Ping Ma, Ling Yuan, Shumin Jia, Ziying Zhou, Duojie Xu, Shicong Huang, Fandi Meng, Zhe Zhang, Yi Nan

**Affiliations:** ^1^ Pharmacy Department, General Hospital of Ningxia Medical University, Yinchuan, Ningxia Hui Autonomous Region, China; ^2^ College of Pharmacy, Ningxia Medical University, Yinchuan, Ningxia Hui Autonomous Region, China; ^3^ Key Laboratory of Ningxia Minority Medicine Modernization Ministry of Education, Ningxia Medical University, Yinchuan, Ningxia Hui Autonomous Region, China; ^4^ Department of Chinese Medical Gastrointestinal, China-Japan Friendship Hospital, Beijing, China

**Keywords:** *Lonicerae Japonicae Flos*, cancer, active ingredients, antitumor mechanisms, pharmacokinetics, quality control, clinical applications, medicine food homology

## Abstract

*Lonicerae Japonicae Flos* (LJF, called Jinyinhua in China), comes from the dried flower buds or flowers to be opened of *Lonicera japonica* Thunb. in the Lonicera family. It has a long history of medicinal use and has a wide range of application prospects. As modern research advances, an increasing number of scientific experiments have demonstrated the anticancer potential of LJF. However, there is a notable absence of systematic reports detailing the anti-tumor effects of LJF. This review integrates the principles of Traditional Chinese Medicine (TCM) with contemporary pharmacological techniques, drawing upon literature from authoritative databases such as PubMed, CNKI, and WanFang to conduct a comprehensive study of LJF. Notably, a total of 507 compounds have been isolated and characterized from the plant to date, which include volatile oils, organic acids, flavonoids, iridoids, triterpenes and triterpenoid saponins. Pharmacological studies have demonstrated that LJF extract, along with components such as chlorogenic acid, luteolin, rutin, luteoloside, hyperoside and isochlorogenic acid, exhibits potential anticancer activities. Consequently, we have conducted a comprehensive review and summary of the mechanisms of action and clinical applications of these components. Furthermore, we have detailed the pharmacokinetics, quality control, and toxicity of LJF, while also discussing its prospective applications in the fields of biomedicine and preventive healthcare. It is hoped that these studies will provide valuable reference for the clinical research, development, and application of LJF.

## 1 Introduction

Cancer, a significant global public health challenge, continues to impose a substantial burden on human lives. According to the International Agency for Research on Cancer (IARC), the number of new cases worldwide in 2020 has risen to 19.3 million, with an estimated 10 million deaths resulting from it (1). Among these, breast, lung, colorectal, prostate, and stomach cancers are responsible for the highest number of cancer-related deaths. Cancer has emerged as the primary cause of mortality, significantly hindering the improvement of life expectancy. Despite the availability of diverse treatment options such as surgery, radiotherapy, chemotherapy, immunotherapy, and targeted therapy, the efficacy of these treatments remains inadequate, with challenges like toxic side effects, drug resistance, tumor recurrence, and metastasis (2). Thus, there is an urgent necessity to explore and develop novel therapeutic approaches and medications to more effectively combat cancer and safeguard human health.

In recent years, numerous complementary and alternative therapies for cancer treatment have emerged. Among these, Traditional Chinese Medicine (TCM) has gained prominence as a significant option due to its low toxicity, safety, and high efficacy (3, 4). As a core component of TCM, Chinese herbs with medicinal and edible value (5, 6), offer advantages such as multi-component, multi-target, and multi-pathway anti-tumor effects. Furthermore, they can integrate medicinal properties with nutritional benefits, thereby providing a unique approach to food therapy that aims to restore the balance of Yin and Yang in the body and enhance overall resistance. Currently, the extracts and active monomer components from various medicinal and edible Chinese herbs have demonstrated anticancer effects. Their integration with surgical resection, chemotherapy, radiotherapy, and targeted therapy not only enhances the efficacy of drug therapy, but also effectively mitigates multi-drug resistance and reduces adverse reactions, thereby significantly improving the quality of life for patients (7). Overall, the role of medicinal and edible herbs in cancer treatment is becoming increasingly significant, offering patients more treatment options and hope.

Lonicerae Japonicae Flos (LJF) is a renowned Chinese herbal medicine, first documented in Shen Nong’s Herbal Classic (Shennong Bencao Jing), where it is classified as a top-quality herb. LJF has a sweet and cold taste and is known for its functions in clearing heat and detoxification, as well as antibacterial and anti-inflammatory. Clinically, it is frequently used to treat conditions such as carbuncles, pustules, throat pain, erysipelas, heat-induced blood dysentery, colds caused by wind heat, and fevers. Due to its therapeutic properties, it has earned the reputation of being a Chinese medicine antibiotic (8, 9).

To date, 507 compounds have been isolated and identified from LJF, including volatile oils, organic acids, flavonoids, iridoids, triterpenes and triterpenoid saponins. Modern pharmacological studies have demonstrated that LJF exhibits a range of effects, including antibacterial, anti-inflammatory, antiviral, liver-protective, intestinal-regulating, anti-depressive, antioxidant, anti-allergic, hypolipidemic, hypoglycemic, and immune-regulatory properties (10–12). Notably, LJF has also shown significant anti-tumor activity. Numerous studies indicate that LJF extract and its active components, such as chlorogenic acid, luteolin, rutin, luteoloside, hyperoside and isochlorogenic acid, exhibit inhibitory effects on various cancers, including liver (13), pancreatic (14), and lung cancers (15). Its mechanism of action mainly includes inhibiting cell proliferation, inducing cell apoptosis, blocking cell cycle, inhibiting cell metastasis, regulating inflammation and immune function, and activating related signaling pathways. In clinical practice, LJF is frequently utilized as an adjunct in cancer treatment to mitigate the side effects of therapies and enhance therapeutic efficacy. Furthermore, in the biomedical field, LJF is also employed in the production of nanoparticles and in the research and development of photosensitizers, thereby expanding its potential applications in cancer treatment.

While previous reviews have highlighted the anti-tumor properties of LJF, a comprehensive systematic review examining the specific anti-tumor effects of LJF extract and its active components has yet to be conducted. Therefore, this review systematically summarizes the mechanisms of action and clinical applications of LJF extract and its active components across various cancers, drawing upon the principles of TCM and modern pharmacological techniques. Additionally, the review analyzes pharmacokinetics, toxicology, and quality control, identifying the limitations associated with the clinical application of LJF. Furthermore, this paper discusses the potential value of LJF in the biomedical field and in preventive health care, aiming to promote its broader application and development in anti-tumor therapies.

## 2 Anti-cancer theory of TCM

TCM has a long-standing history in cancer treatment, with its fundamental therapeutic principle centered on enhancing the body’s resistance and eliminating pathogenic factors (16). In the following sections, I will elaborate on LJF’s TCM anti-cancer theory from these two perspectives and explore its compatibility with contemporary medical theories.

### 2.1 Strengthening body resistance

According to the Huangdi Neijing (The Yellow Emperor’s Inner Classic), TCM posits that the development of cancer is closely associated with a deficiency of healthy qi and the invasion of pathogenic factors within the human body. The intrusion of pathogenic factors disrupts the delicate balance of Yin and Yang, leading to dysfunction in the zang-fu organs and impairing the flow of Qi-Blood-Body fluid, which can result in various pathological changes throughout the body or in specific localized areas. Therefore, the principle of TCM (17, 18) in the treatment of cancer focuses on strengthening the body’s foundational health, promoting blood circulation, and removing blood stasis. This approach aims to achieve a balance between Yin and Yang, enhance the body’s resistance, and suppress tumor growth.

Modern medicine posits that immunity plays a crucial role in enhancing the body’s resistance and maintaining its relative stability. The development of cancer is closely associated with a diminished function of the human immune system (19). Consequently, the deficiency of healthy qi in TCM is theoretically regarded as analogous to low immunity in modern medicine. The TCM concept of strengthening healthy qi to eliminate pathogenic factors is highly related to the strengthening of immunity emphasized by modern medicine. Recent pharmacological studies have demonstrated (20) that LJF can modulate the body's immune function through various targets and pathways, thereby enhancing the ability to resist cancer invasion. Therefore, the principles of strengthening health qi and eliminating pathogenic factors, which are emphasized in TCM, can serve as a guiding theory for the anticancer effects of LJF.

### 2.2 Clearing heat and detoxification, eliminating pathogenic factors

According to the theory of TCM, cancer is considered a disease resulting from the accumulation of heat toxins and the stasis of qi and blood within the body. Similar to inflammatory factors, pathogenic heat and toxins are implicated in the etiology of cancer and can facilitate its progression. As a traditional Chinese herbal medicine, LJF exhibits numerous pharmacological effects, including antioxidant, anti-inflammatory, antibacterial, and antiviral properties, which are linked to its heat-clearing and detoxifying capabilities (21, 22).

In clinical practice, patients with middle to advanced stages of cancer frequently experience symptoms such as localized lumps and burning pain. Consequently, a primary treatment approach involves the use of heat-clearing and detoxifying medications (23–25). By effectively eliminating heat and toxins from the body, these treatments can significantly alleviate the pain experienced by cancer patients. This approach not only aligns with the theoretical principles of TCM but also supports its practical application in clinical settings.

## 3 Network pharmacological analysis

To explore the link between LJF and cancer, we conducted an exhaustive search of the active ingredients and their associated targets with the help of Traditional Chinese Medicine Systems Pharmacology Database and Analysis Platform (TCMSP) (https://old.tcmsp-e.com/tcmsp.php). The active ingredients were required to satisfy two key conditions: oral bioavailability (OB) ≥ 30% and drug likeness (DL) ≥ 0.18. Then we used the Uniprot database (https://www.uniprot.org/) to standardize the target names. Finally, the drug-component-target network diagram was constructed by Cytoscape 3.9.0 software, and Kyoto Encyclopedia of Genes and Genomes (KEGG) enrichment analysis was performed using the DAVID database (https://david.ncifcrf.gov/). The top 30 key signaling pathways were screened based on p-values and histograms were plotted with the help of the bioinformatics online platform. The smaller the p-value, the more significant the enrichment of the pathway.

**Figure 1:**
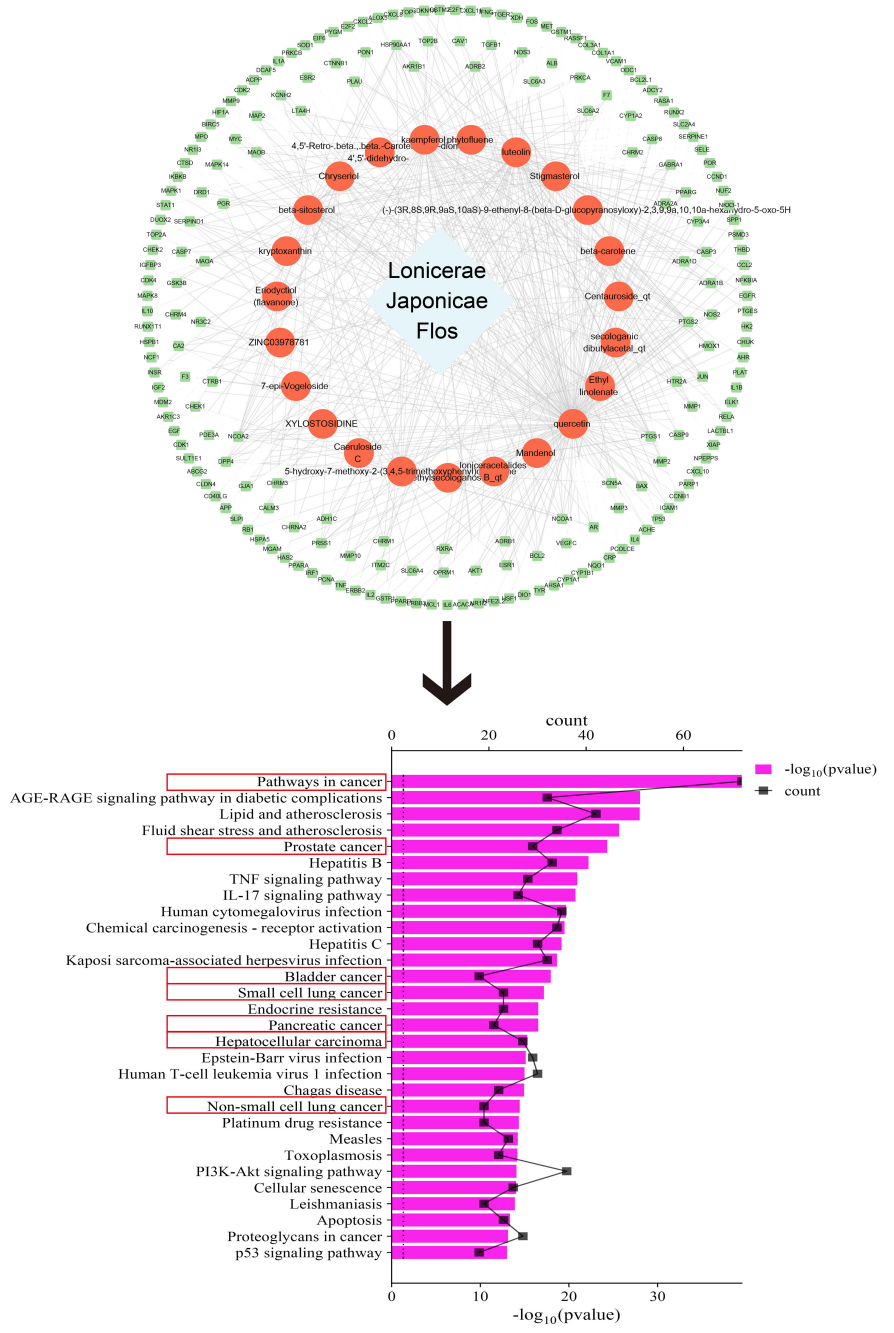
Target acquisition and related pathway screening of LJF.

As shown in **Figure 1**, after screening, we obtained 23 active ingredients from LJF, involving 199 targets. After KEGG analysis, we successfully enriched 176 signaling pathways, among which 7 of the top 30 signaling pathways were closely related to tumors.

## 4 Active ingredients

**Figure 2:**
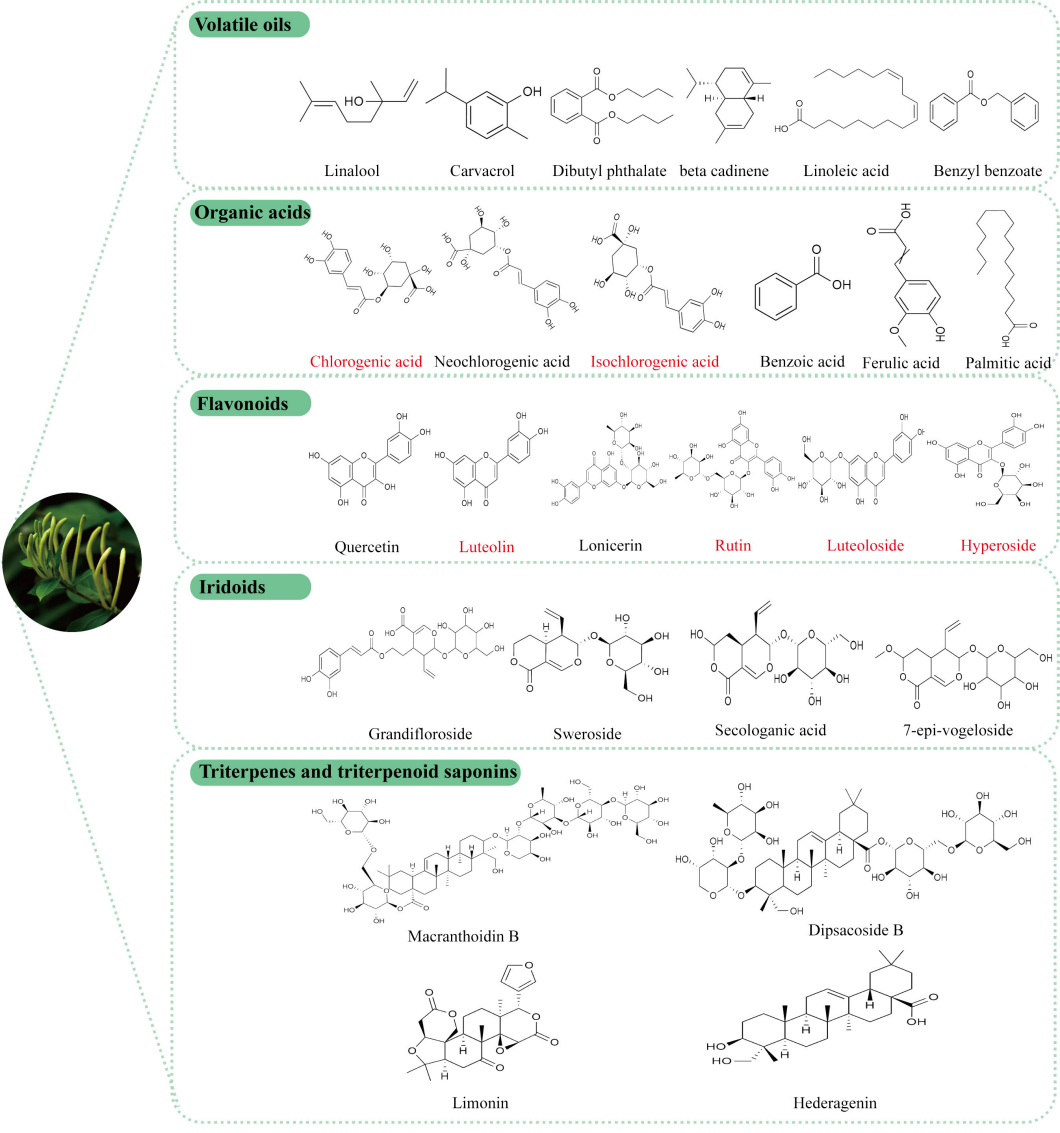
Structural formula of the active components of LJF, the red label is the main representative component of LJF’s anti-tumor effect.

In recent years, comprehensive studies on the active components of LJF have elucidated its unique pharmacological effects and potential applications. Phytochemical analyses have identified various bioactive compounds in LJF, including volatile oils, organic acids, flavonoids, iridoids, triterpenes and triterpenoid saponins (26, 27). These active ingredients form a robust basis for the medicinal effects attributed to LJF. The specific chemical structure formula is shown in **Figure 2**.

### 4.1 Volatile oils

LJF, known for its distinct aromatic scent, contains a variety of volatile oil components, including acids, aldehydes, alcohols, ketones, and their esters. These components may vary depending on geographical location. Through detailed identification (28, 29), it has been found that in the Ningxia Province region of China, the three main volatile oil components in LJF are linalool (13.59%), carvacrol (7.67%), and dibutyl phthalate (7.54%), all of which have demonstrated anticancer properties (30–32). In Hunan Province (33), the primary components of LJF consist of n-hexadecanoic acid, linoleic acid, and α-curcumene. Linoleic acid, an essential nutrient, not only enhances anti-tumor immunity (34), but also plays a role in regulating glucose homeostasis.

### 4.2 Organic acids

The common organic acids, including chlorogenic acid, neochlorogenic acid, and isochlorogenic acid, have been identified to possess various pharmacological activities such as anti-inflammatory (35), anti-tumor (36), antibacterial (37), antioxidant (38), hypoglycemic (39), neuroprotective (40). Additionally, a novel organic acid compound, (2S,7R,2′R)-2-(3-hydroxy-5-methyl-4-oxo-3,4-dihydro-2H-pyran-6-yl)-3-((2-hydroxypropanoyl) oxy) propanoic acid, has recently been discovered to exhibit hepatoprotective effects (41).

### 4.3 Flavonoids

Flavonoids are compounds found in various herbal plants and have been shown to exhibit a range of biological activities that offer protective effects on different organs (42). Quercetin, luteolin, luteoloside, hyperoside, lonicerin, and rutin, which are extracted from LJF, are examples of flavonoids with proven antibacterial (43), anti-inflammatory (44), antioxidant (45), immune-regulating (46) and anticancer activities (47). The concentration of flavonoids in LJF is closely linked to its growth stage (48), with studies indicating that the content initially increases, then decreases, peaking at the white alabastrum stage (49). Furthermore, research has demonstrated that luteoloside specifically possesses antidepressant properties (50).

### 4.4 Iridoids

Iridoids are the most abundant compounds in LJF, with secoiridoid glycosides making up more than half of them. These include secologanic acid, sweroside, and loniaceticiridoside. Sweroside has been found to partially reduce hepatic steatosis by activating AMPK/mTOR-mediated autophagy in mice (51), as well as protect against LPS-induced ALI by suppressing inflammation (52). Recent studies have shown that secoiridoid glucosides and their derivatives extracted from LJF buds, such as secoxyloganin and dimethylsecol-ologanoside, have inhibitory effects against influenza A (53). Additionally, two new iridoids were isolated from LJF leaves by Yu J et al. (41), namely (1R,5R,9S)-5-epi-sweroside and rel-(1R,5R,9R)-1-O-β-D-glucopyranosyl-9-vinyl-2-oxabicyclo[4.3.0]non-3-en-11-one. (1R,5R,9S)-5-epi-sweroside showed weaker antioxidant activity than quercetin and a slightly lower hepatoprotective effect than magnesium isoglycyrrhizinate.

### 4.5 Triterpenes and triterpenoid saponins

More than 35 triterpenoids and triterpenoid saponins have been identified in LJF (54). Ursolic acid has been found to enhance the antitumor effects of sorafenib in human cancers through mechanisms related to Mcl-1-dependent apoptosis and SLC7A11-related iron apoptosis (55). *In vitro* studies have shown that hederagenin can inhibit ovarian cancer cell proliferation by modulating mitochondrial translocation and apoptosis (56). Daucosterol has been reported to induce autophagy-dependent apoptosis in prostate cancer cells via activation of the c-Jun N-terminal kinase (JNK) signaling pathway (57). Additionally, loniceroside A, loniceroside B, loniceroside C, loniceroside D, and loniceroside E (58, 59), all classified as saponins, with loniceroside C demonstrating significant anti-inflammatory activity in a croton oil-induced ear edema assay in mice (60).

## 5 Anti-tumor mechanism

**Figure 3:**
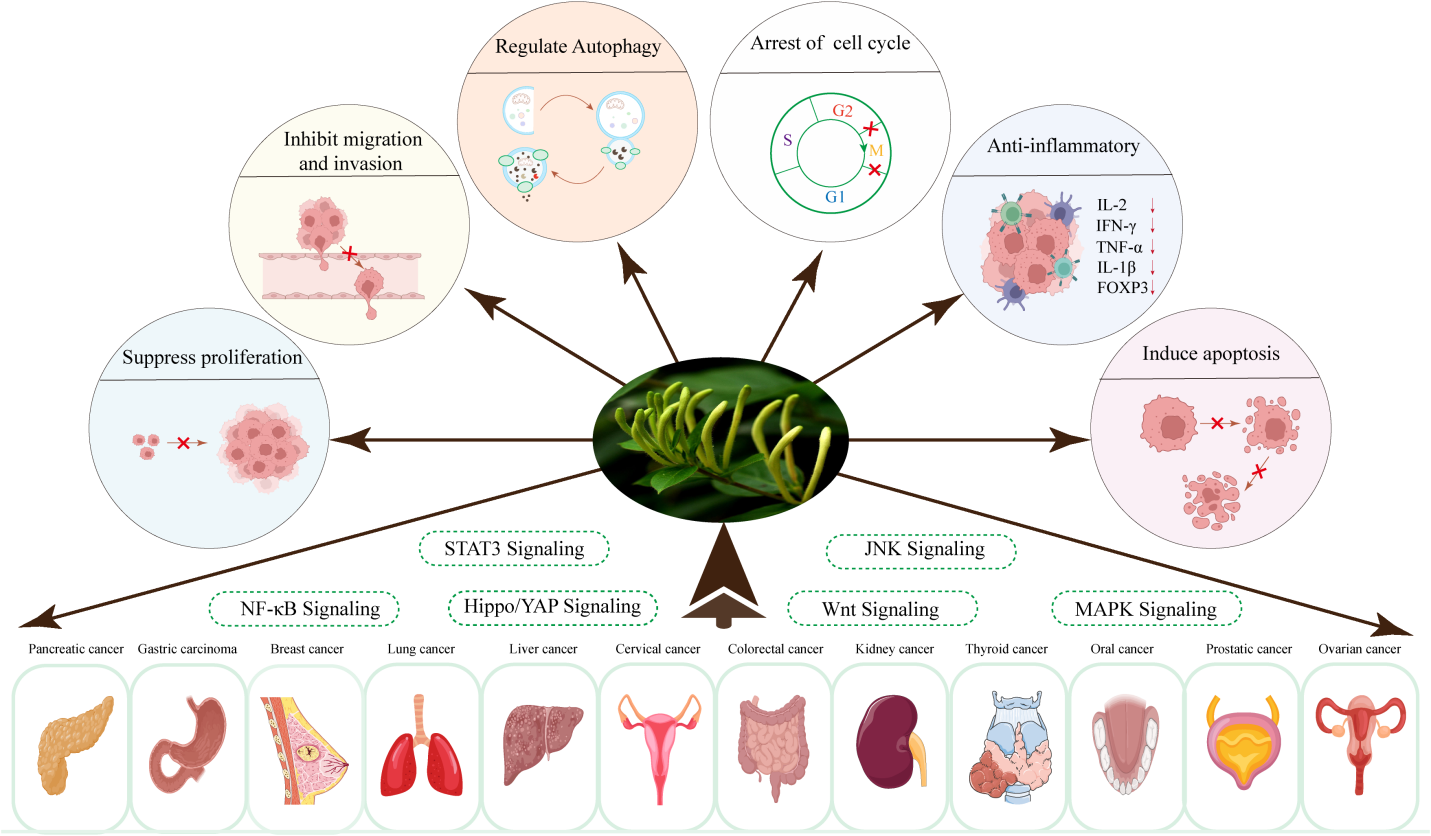
Types and phenotypes of LJF anti-tumors.

**Figure 4:**
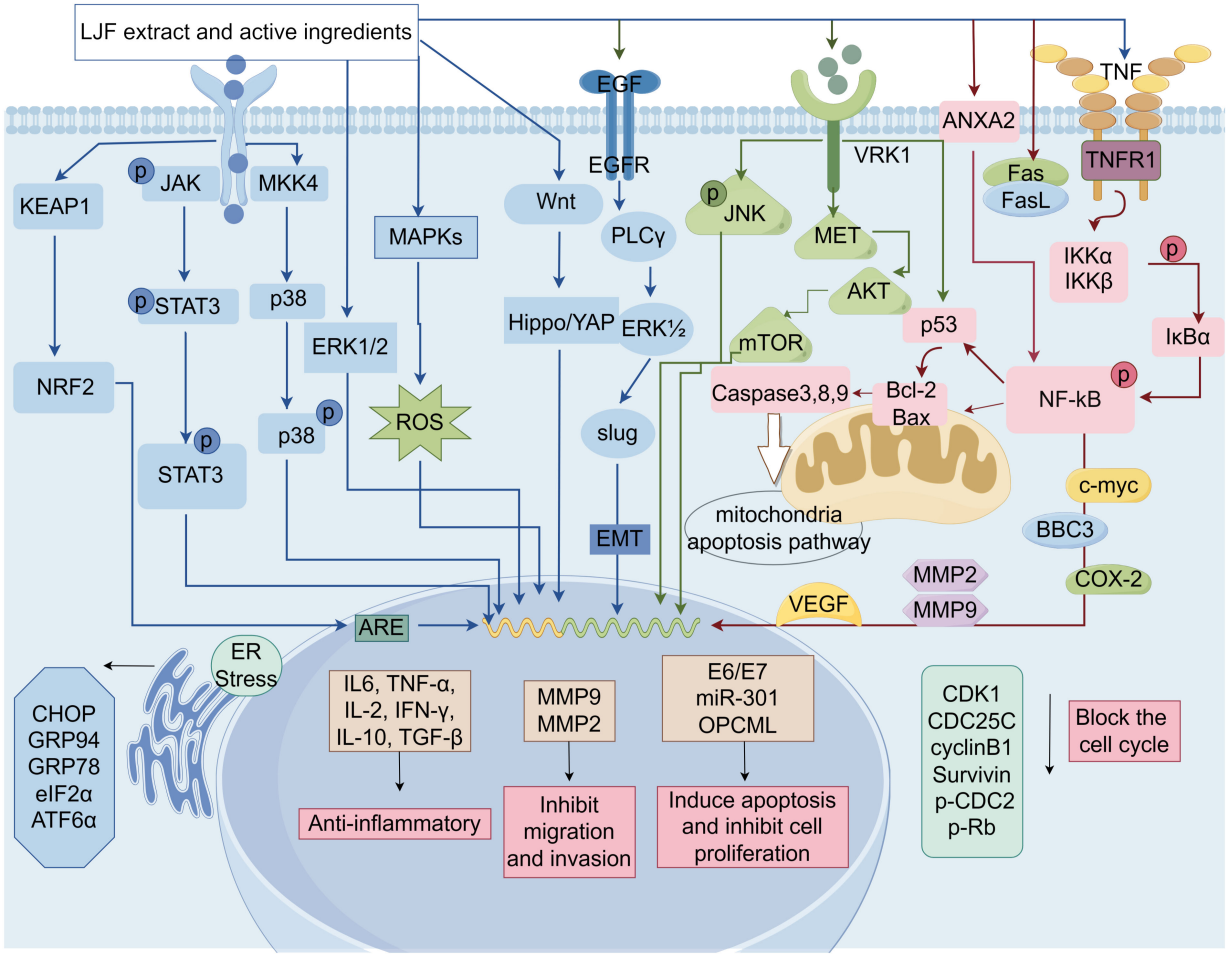
The molecular mechanisms of LJF and its active compounds for cancer treatment (Created via Figdraw).

Over the past few decades, researchers have concentrated on investigating the effects of LJF and its active compounds on various cancers. The comprehensive study of LJF extract and its active components is crucial for elucidating the anti-tumor mechanisms associated with this plant. As research has progressed, the anti-tumor mechanisms of LJF have gradually been unveiled. Numerous studies indicate that LJF extract, along with its active ingredients such as chlorogenic acid, luteolin, rutin, luteoloside, hyperoside and isochlorogenic acid, possesses the capability to combat various malignant tumors (**Table 1**). The mechanisms of action include inhibiting cancer cell proliferation, blocking the cell cycle, inducing apoptosis, and inhibiting cell migration. Additionally, LJF also has anti-inflammatory, regulating immune function, autophagy, and activating related signaling pathways. The specific action pathways and molecular mechanisms are illustrated in **Figures 3** and **4**.

**Table 1:** Antitumor effects of LJF and its active components.

Type of Cancer	Ingredient	Cell Type/Animal Model	Phenotype	Target of Action	Mechanisms of action	Reference
Liver cancer	3,4-di-Ocaffeoylquinic acid isobutyl ester	HepG2, Huh7	Proliferation, migration	Inhibition of cell proliferation and migration through regulation of YAP/CTGF	YAP/CTGF	(61)
Liver cancer	Protocatechuic acid	HepG2	Proliferation	Induction of hepatocellular carcinoma cell death in a JNK-dependent manner	JNK	(62)
Liver cancer	Japoflavone D	SMMC-7721	Apoptosis	Induces apoptosis by up-regulating the AKT/mTOR signaling pathway, ERK activity, and down-regulating the KEAP1/NRF2/ARE signaling axis	AKT/mTOR, ERK, KEAP1/NRF2/ARE signaling axis	(63)
Liver cancer	Polyphenolic extract	HepG2	Apoptosis	Induction of apoptosis in hepatocellular carcinoma cells by inhibition of PI3K/Akt and activation of MAPK	PI3K/Akt, MAPKs	(64)
Liver cancer	3,5-dyhydroxy-7-methoxyflavone	HepG2, SMMC-7721	Apoptosis	Induction of apoptosis through the intrinsic apoptotic pathway	Bcl-2/Bax	(13)
Liver cancer	Luteoloside	HepG2	Proliferation, apoptosis	Anti-proliferative and pro-apoptotic effects through activation of JNK and cell cycle blockade to G2/M phase	JNK	(65)
Hepatocellular carcinoma	Luteoloside	HCC	Proliferation, metastasis	Inhibition of HCC cell proliferation, invasion and metastasis by inhibiting NLRP3 inflammatory vesicles	NLRP3	(66)
Colon cancer	Hydnocarpin	HEK293, SW480	Proliferation	Inhibit cell proliferation by regulating Wnt signaling pathway	Wnt/β-catenin signaling pathway	(67)
Colon cancer	Chlorogenic acid	HCT116, HT29	Proliferation, anti-inflammatory	Inhibits cell proliferation by inactivating ERK and inducing ROS generation	ROS, ERK	(68)
Colon cancer	Chlorogenic acid	Caco-2	Proliferation	Inhibits cell proliferation by regulating caspase-3 expression	Caspase-3	(69)
Gastric cancer	Chlorogenic acid	male F344 rats	Proliferation	Inhibition of cell proliferation and reduction of adenomatous hyperplasia	–	(70)
Gastric cancer	HGC27, MKN45 and Luteoloside7, MKN45 and SGC7901	HGC27, MKN45, SGC7901	Proliferation, metastasis	Regulation of cell proliferation, apoptosis, autophagy, invasion and tumorigenesis through the MET/AKT/mTOR axis	MET/AKT/mTOR axis	(71)
Lung cancer	Polyphenolic compounds	A549	Apoptosis	Induces apoptosis by regulating the protein expression levels of caspases, poly-(ADP-ribose) polymerase and the B-cell lymphoma-2-associated X-protein/B-cell lymphoma-extra large ratio	AKT/caspase cascade	(15)
Lung cancer	Alcohol extract	CH27	Apoptosis	Induces apoptosis by affecting mitochondrial function, endoplasmic reticulum stress, and ROS generation	PDI/DJ-1/ATP synthase/heat shock protein 70/chaperonin	(72)
Human non-small cell lung cancer	Luteoloside	A549, H292	Proliferation, EMT	Inhibits cell proliferation by inducing autophagy and activating PI3K/AKT/mTOR/p70S6K signaling	PI3K/AKT/mTOR/p70S6K signaling	(73)
Human lung squamous carcinoma	Lonicera japonica extract	CH27	Apoptosis	Induces apoptosis by activating the P38-related pathway and affecting the expression and distribution of heat shock protein 27	Caspase-3/AIF/heat shock protein 27/P38	(74)
Human nasopharyngeal carcinoma	Luteoloside	NPC-039, NPC-BM	Proliferation, apoptosis	Induction of apoptosis through activation of exogenous and endogenous apoptotic pathways and activation of AKT signaling	Exogenous and endogenous apoptotic pathways. AKT signaling	(75)
Kidney cancer	Chlorogenic acid	A498	Proliferation, apoptosis	Inhibits proliferation and promotes apoptosis by activating the PI3K/Akt/mTOR signaling pathway	PI3K/Akt/mTOR signaling pathway	(76)
Colorectal cancer	Luteoloside	HCT15, RKO, HCT116, LoVo, HCoEpiC	Proliferation	Inhibits cell proliferation by decreasing the expression of the nuclear antigen KI67 and inhibiting the expression of CDC25A	CDC25A	(77)
Oesophageal carcinoma	Chlorogenic acid	CE81T-M4	Metastasis	Inhibits cell migration and invasion by inhibiting the EGFR/p-Akt/Snail pathway	EGFR/p-Akt/Snail signaling pathway	(78)
Oesophageal carcinoma	Chlorogenic acid	ESCC	Proliferation,metastasis	Inhibition of proliferation and migration through down-regulation of BMI1 and SOX2 expression	BMI1/SOX2	(79)
Breast cancer	Chlorogenic acid	4T1, EMT6, BT-549, MDA-MB-231, Female SPF-Balb/c mice	Proliferation, migration, apoptosis	Inhibits cell proliferation, migration and induces apoptosis by inhibiting the expression of VEGF, EGF, IL-10, TGF-β and CD34	Ligands of RTKs, inflammatory factors	(80)
Breast cancer	Chlorogenic acid	4T1 mouse BC	Apoptosis	Induces apoptosis by down-regulating Bcl-2, up-regulating p53, Bax, caspase-3	P53/Bax/Bcl-2/caspase-3 pathways	(81)
Ovarian cancer	Chlorogenic acid	OVCA43, SKOV3	Migration, invasion, apoptosis	Inhibits cell migration by activating the mitochondria-mediated intrinsic apoptotic pathway	MMP, EMT, NF-κB	(82)
Cervical cancer	Luteoloside	Hela	Proliferation, apoptosis	Induces apoptosis by regulating the p53/mTOR signaling pathway, both intrinsic and extrinsic apoptotic pathways	P53/mTOR signaling pathway	(83)
Cervical cancer	Exosomal miR2911	HEK293T	Proliferation, apoptosis	Inhibits proliferation and induces apoptosis by targeting the E6/E7 genes of HPV16/18	E6/E7-p53/Caspase3 axis	(84)
Acute myeloid leukemia	Sweroside	HL-60	Proliferation, apoptosis	Induced cell cycle arrest at the S and G2/M phases by reducing the levels of cyclin D1, CDK4, CDC2, and CDC25, while increasing the expression of p53 and p21	CyclinD1/CDK4/CDC2/CDC25/p53/p21	(85)
Leukemia	Polyphenols	U937	Apoptosis	Induced apoptosis by upregulation of DR4 and Fas, and further it is augmented by suppression of XIAP	DR4/Fas/XIAP	(86)
Melanoma	Ethanolic extract	B16F10	Immunomodulation, proliferation	Suppresses tumor growth by inhibiting STAT3 signaling and remodeling the immune microenvironment	STAT3	(20)
Thyroid cancer	Saponins	TPC-1	Proliferation, invasion, apoptosis	Inhibits cell proliferation, invasion, and induces apoptosis by inhibiting the SHP2/Ras/MAPK signaling pathway	SHP2/Ras/MAPK	(87)
Burkitt’s lymphoma	Lonicera japonica extract	CD19 CAR-T	Proliferation, apoptosis	Induces apoptosis and inhibits proliferative capacity *in vitro* by inhibiting PD-1 and elevating granase B	PD-1/granase B	(88)
S180 sarcoma	Polysaccharide	S180 solid tumor model mice	Proliferation	The mechanism of tumor growth inhibition may be related to the regulation of the Bcl-2/Bax apoptotic pathway and the promotion of TNF-α secretion	Bcl-2/Bax apoptotic pathway, TNF-α	(89)
Oral squamous cell carcinoma	Neochlorogenic acid	A-253, HSC-4, CAL-27, SCC-4	Apoptosis	Induces apoptosis and cell cycle arrest by regulating CDK1/catenin B1 expression and the mitochondria-mediated TOP2A-dependent apoptosis pathway	TOP2A, CDK1/cyclin B1	(36)
Oral cancer	Luteoloside	FaDu, HSC-3, CA9-22	Proliferation, metastasis	Reduces cancer metastasis and blocks cell proliferation by decreasing p38 phosphorylation and downregulating MMP-2 expression	p-p38/MMP-2	(90)
Neuroblastoma	Luteoloside	SH-SY5Y, SK-N-AS	Proliferation	Inhibits cell proliferation by decreasing the expression of G0/G1 phase-associated proteins D1, CDK4 and C-myc	p38/MAPK pathway	(91)

### 5.1 Inhibition of cancer cell proliferation

Cancer arises from the abnormal proliferation and differentiation of normal cells, influenced by both internal and external factors. This uncontrolled proliferation is a defining characteristic of tumor cells and is intrinsically linked to the cancer development process. Consequently, an essential aspect of cancer treatment is the effective inhibition of the excessive growth and proliferation of tumor cells (92, 93).

Several studies have shown that LJF extract and its active ingredients have the ability to exert anti-cell proliferation in specific cancers. Chlorogenic acid (80) regulates cell proliferation, apoptosis and tumor-microenvironment by down-regulating the expression of RTK ligands and inflammatory factors, and has shown outstanding therapeutic effect on breast cancer. In addition (94), chlorogenic acid binds to annexin A2, resulting in decreased expression of downstream NF-κB anti-apoptotic gene, thereby inhibiting the growth of human lung cancer A549 cells. In addition, chlorogenic acid (95) has the function of activating ERK1/2. This mechanism plays a significant inhibitory role in the proliferation of osteosarcoma U2OS cells.

Han et al. demonstrated that luteolin inhibits the proliferation and induces apoptosis of prostate cancer (PCa) cells by triggering DEDD2 expression and down-regulating miR-301. As a prognostic marker, miR-301 may represent a novel approach for PCa treatment (96). Furthermore, luteolin (97) has been shown to reduce the expression level of VRK1, activate the p53 signaling pathway, induce G2/M cell apoptosis and cell cycle arrest, and effectively inhibit the proliferation of high-grade serous ovarian cancer (HGSOC) cells. More importantly, luteolin (98) was found to enhance the expression of the OPCML gene and promote its demethylation process, which contributes to the inhibition of proliferation in breast cancer MDA-MB-231 cells. The OPCML gene has been recognized by the scientific community as a crucial tumor suppressor gene, essential for maintaining normal cell function, differentiation, and development. Its abnormal expression can lead to uncontrolled cell growth.

Luteolin has been shown to possess significant pharmacological effects in the diagnosis and treatment of nasopharyngeal carcinoma. It can precisely regulate both exogenous and endogenous apoptotic pathways, as well as AKT signal transduction pathways, thereby effectively inhibiting the proliferation and inducing apoptosis in nasopharyngeal carcinoma cell lines, such as NPC-039 and NPC-BM (75). Furthermore, this compound also can reduce mitochondrial membrane potential and diminish the production of reactive oxygen species, subsequently activating caspase-3 and caspase-8 (83). This activation alters the nuclear morphology of cells and causes DNA damage. Collectively, these effects demonstrate a pronounced inhibitory impact of luteolin on HeLa cell proliferation in cervical cancer and initiate the apoptotic process.

The exosomal miR2911 derived from LJF (84) has been reported to exhibit significant antitumor effects. Its mechanism of action involves targeting the E6/E7 genes of HPV16/18, thereby effectively inhibiting the proliferation of cervical cancer cells. Concurrently, experimental results demonstrated that at a concentration of 1 mg/mL, the polysaccharide extract of LJF (14) significantly inhibited the growth of BxPC-3 and PANC-1 pancreatic cancer cells, with inhibition rates reaching 66.7% and 52.1%, respectively. These findings not only provide robust evidence for the anti-proliferative properties of LJF extract but also suggest new directions and ideas for the development of novel therapeutic strategies.

### 5.2 Induction of cell cycle arrest

The cell cycle is the process through which cells progress from the completion of one division to the end of the next, and it is divided into two main phases: interphase and division. The progression of the cell cycle is primarily regulated by cyclin-dependent kinases (CDKs), cyclins, and endogenous CDK inhibitors (CKIs), which interact to influence cell proliferation, growth, and repair. Regulation of the cell cycle is a complex and delicate process. any abnormalities can lead to uncontrolled cell proliferation and the development of cancer (99, 100). Consequently, adjusting cell cycle distribution and inducing cell cycle arrest are considered effective strategies for cancer treatment.

A study indicated that LJF polyphenol extract (64) induced apoptosis in hepatocellular carcinoma cells by decreasing the expression of CDK1, CDC25C, and Cyclin B1, thereby obstructing the cell cycle at the G2/M phase. When compared to control data (12.55 ± 3.14%), treatment with 600 and 800 µg/ml of polyphenol extract significantly increased the percentage of cells in the G2/M phase to 25.37 ± 1.67% and 27.13 ± 2.12%, respectively.

An experimental study demonstrated that rutin mediates cell death in human cervical cancer Caski cells by down-regulating the expression levels of CDK4, Cyclin D1, Notch-1, and Hes-1, effectively blocking the cell cycle at the G0/G1 phase (101). Furthermore, Huang et al. found (102) that luteolin inhibited the cell cycle in the S phase and induced apoptosis in breast cancer MDA-MB-231 cells by down-regulating the expression of hTERT, Cyclin D1, and Survivin, while simultaneously increasing the expression level of p21. The proposed mechanism of action may involve the inhibition of the NF-κB-c-Myc axis, leading to reduced hTERT expression. Additionally, luteolin has the capacity to modulate mitochondrial function, thereby facilitating apoptotic cell death. Cai et al. reported (103) that luteolin treatment of lung cancer A549 cells resulted in a significant increase in the proportion of G2 phase cells and a marked decrease in the expression of G2 phase cell cycle-related proteins (cyclin A, p-CDC2, and p-Rb), ultimately inducing apoptotic cell death.

Moreover, Lei et al. found (77) that cynaroside significantly inhibited colorectal cancer cell proliferation and colony formation *in vitro* by down-regulating CDC25A, which induced G1 cell cycle arrest. The inhibitory effect of the active ingredient luteoloside on the growth of liver cancer cells is mainly due to G2/M phase arrest and reactive oxygen species (ROS) production. In addition, luteoloside also increased the phosphorylation of JNK (65).

### 5.3 Induction of tumor cell apoptosis

Apoptosis (104) is a mode of programmed death that is genetically regulated and can be involved in a variety of pathological processes. During cell death, significant changes in cell morphology occur. These changes include reduction in cell size, fragmentation of the nucleus, and the appearance of eosinophilic vesicles in the cytoplasm. These features can be used to identify the type of apoptosis (105, 106). Apoptosis has long been recognized as an important mechanism for preventing the emergence of tumors. Therefore, killing cancer cells through the apoptotic pathway has been the mainstay of clinical cancer therapy.

In recent years, numerous studies have focused on inducing apoptosis by targeting apoptosis-related genes in the pursuit of improved cancer treatments. The anti-apoptotic protein Bcl-2 and the pro-apoptotic protein Bax are pivotal regulatory genes in the apoptosis process, as they regulate the release of substances such as cytochrome C through the mitochondrial pathway, thereby mediating cell survival or death. Consequently, effectively targeting the pro-apoptotic effects of Bcl-2 family members may represent a promising strategy for cancer therapy.

Qi et al. (107) found that LJF polysaccharide extract promoted apoptosis in triple-negative breast cancer cells by regulating the Bcl-2/Bax ratio. Hyperoside can inhibit the proliferation and promote apoptosis of human pancreatic cancer cell lines PANC-1 and BxPC-3 by up-regulating the Bax/Bcl-2 and Bcl-xl ratios, and down-regulating NF-κB as well as survivin, c-Myc, cyclin D1, and COX-2 (108). In particular, NF-κB plays an important role in regulating the level or localization of Bcl-2 family proteins in cells. This was confirmed in the study of chlorogenic acid against liver cancer *in vitro*. The specific mechanism involves chlorogenic acid (109) activating the mitochondrial apoptotic pathway in Hep-G2 and Ha-7 cells by inhibiting the non-classical NF-κB signaling pathway and up-regulating BBC3, a member of the BH3-only pro-apoptotic subclass. The BH3 domain is regarded as an essential structural component in the apoptosis process, playing a significant role in pro-apoptotic activity.

All apoptotic signaling pathways have been reported to be activated by cysteinyl aspartate-specific proteinase (110). These enzymes function by specifically targeting cysteine residues in proteins and cleaving peptide bonds following aspartic acid residues. Consequently, the activation of caspase to induce apoptosis has emerged as a significant strategy in tumor therapy. Through a literature search, we identified various active ingredients in LJF that possess the potential to activate caspase. hyperoside and rutin (111) have been shown to induce apoptosis in HT-29 human colon cancer cells via the activation of cleaved caspase-3, caspase-8, and caspase-9. This phenomenon is mediated through the mitochondrial intrinsic apoptosis pathway, offering novel insights for cancer therapy. Additionally, an *in vivo* study revealed (81) that treatment of 4T1 breast cancer mice with chlorogenic acid significantly increased caspase-3 gene expression, highlighting a key mechanism underlying the pro-apoptotic effect of chlorogenic acid.

The aforementioned studies have demonstrated that LJF extract and its active ingredients possess the potential to activate the mitochondrial intrinsic apoptotic pathway, exhibiting significant therapeutic promise in cancer treatment. Notably, apoptosis occurs through two primary signaling pathways, the extrinsic/death receptor pathway and the intrinsic/mitochondrial pathway. It has been reported that hyperoside (112) effectively induces apoptosis in human thyroid squamous cell carcinoma SW579 by upregulating the expression of Fas and FasL mRNA while downregulating the expression of survivin. This outcome is partly attributed to the activation of the extrinsic death receptor pathway following the binding of Fas and FasL.

Recent studies have emphasized that oxidative stress-mediated apoptosis represents a promising therapeutic strategy for targeting cancer cells (113). Elevated levels of ROS play a significant role in the induction of apoptosis. In colon cancer investigations, luteolin (114) has been shown to induce apoptotic cell death by enhancing the antioxidant activity of human colon cancer HT-29 cells. The specific mechanisms involved include ROS scavenging and the activation of the MAPK signaling pathway. Meanwhile, Japoflavone D (63), extracted from LJF buds, effectively mitigates cellular damage caused by excessive ROS through the activation of the KEAP1/NRF2/ARE signaling axis, thereby regulating apoptosis. Furthermore, numerous studies have demonstrated that ROS levels correlate with endoplasmic reticulum stress. Notably, Wang et al. showed that (115) luteolin induced lethal stress and ultimately apoptosis in glioblastoma cells by increasing ROS levels within the endoplasmic reticulum.

The endoplasmic reticulum is a crucial organelle within the cell that facilitates the proper folding of newly synthesized proteins through processes such as methylation, hydroxylation, lipidation, and the formation of disulfide bonds (116). However, when a significant accumulation of unfolded or misfolded proteins occurs in the lumen of the endoplasmic reticulum, a cellular stress response known as endoplasmic reticulum stress (ERS) is activated. In response to this stress, the cell initiates the unfolded protein response (UPR). Under conditions of normal homeostasis, the UPR serves as an adaptive response program. However, when the level of stress surpasses the adaptive capacity of the UPR, apoptosis is triggered. Consequently, the precise regulation of ERS is of paramount importance, as it directly influences cell survival or death.

In a recent study, we demonstrated that luteolin (117) derived from LJF induce the upregulation of the UPR pathway through ERS sensors, thereby contributing to the regulation of the apoptotic pathway in adrenal medullary pheochromocytoma PC12 cells. Furthermore, luteolin (118) plays a role in regulating the expression of ERS-associated proteins, including CHOP, GRP94, and GRP78, as well as the cleavage of ATF6α and the phosphorylation of eIF2α. These regulatory processes ultimately trigger ERS, leading to the induction of apoptosis.

### 5.4 Inhibition of cell metastasis

Cancer metastasis, defined as the dissemination of cancer cells from the primary tumor to adjacent tissues and distant organs, is a critical factor in cancer progression and patient mortality (119). Research has identified several mechanisms that significantly contribute to tumor cell migration and invasion, including epithelial-mesenchymal transition (EMT), tumor angiogenesis, the initiation of an inflammatory tumor microenvironment, and apoptosis (120–122). Consequently, inhibiting these pathways may effectively impede the metastasis of cancer cells and slow tumor progression.

Metastasis is a complex process that involves several stages, including invasiveness, introgression, extravasation, and growth in distant organs. Tumor invasion through the extracellular matrix (ECM) is recognized as a critical stage in this progression. Several matrix metalloproteinases, such as MMP-2 and MMP-9, are believed to play direct roles in the migration, invasion, and metastasis of tumor cells, and they are associated with various prognostic factors. Among the active monomer components of LJF, luteolin and luteoloside have demonstrated anti-metastatic effects. Luteolin (123) acts as an anti-metastatic agent by inhibiting the production of MMP-9 and MMP-2, while luteoloside (90) regulates the expression of MMP-2 in human oral squamous cell carcinoma, including FaDu, HSC-3, and CA9-22, and inhibits cell migration and invasion. Additionally, In addition, the ethanol extract mixture of LJF (50μg/mL) also inhibited the migration and invasion of melanoma cells (124). It was found to significantly reduce melanoma cell viability by increasing miR-let-7a/f levels and decreasing the expression of CCR7, MMP-2, MMP-9, p-p38, and p-JNK proteins in melanomainvaded lung tissues.

It is becoming increasingly evident that (120) EMT-a process wherein epithelial cells lose their morphology and function, gradually transforming into mesenchymal-like cells-is associated with tumor recurrence and metastasis. During this transformation, epithelial cells acquire characteristics typical of mesenchymal cells, leading to enhanced motility and migration. Numerous regulatory factors influence EMT, including E-cadherin, transforming growth factor β, the Wnt signaling pathway, the circular RNAs, and various transcription factors (125). Notably, the activation of E-cadherin serves as a marker for the initiation of EMT. In a scientific study, treatment of the triple-negative breast cancer cell line MDA-MB-231 with isochlorogenic acid C (126), sourced from LJF, resulted in a decrease in the expression of mesenchymal markers slug and vimentin, alongside an increase in the expression of the epithelial marker E-cadherin, compared to the control group, indicating a reversal of EMT. Furthermore, isochlorogenic acid C was found to decrease the expression of MMP-9, a key driver involved in ECM degradation.

Tumor angiogenesis plays a crucial role in the metastasis of tumor cells to distant organs. Vascular endothelial growth factor A (VEGFA) serves as a key regulator, stimulating the formation and expansion of tumor blood vessels to supply essential nutrients and oxygen, thereby promoting rapid tumor growth and metastasis. A recent study has demonstrated that luteolin (127), the active compounds found in LJF, exhibit anti-angiogenic properties. Specifically, it can inhibit angiogenesis in gastric cancer by suppressing the secretion of VEGF, which depends on the expression of Notch1, ultimately leading to a reduction in the migration and proliferation of gastric cancer cells. Conversely, rutin (128), another active ingredient in LJF, appears to activate the angiogenic pathway that facilitates the spread of breast tumors to adjacent organs. This occurs because rutin enhances the expression of the pro-angiogenic marker VEGFA while diminishing the expression of the anti-angiogenic marker Thrombospondin 1 in the MDA-MB-231 cell line.

### 5.5 Anti-inflammatory and immunomodulatory effects

There exists a significant connection between the occurrence and development of malignant tumors and the body’s immune defense mechanisms. As tumor cells proliferate, spread, and metastasize, the body’s immune function tends to decline (129). Relevant reports indicate that chronic inflammation plays a crucial role in all stages of tumor development, as well as in the treatment process. It serves not only as a factor that induces tumorigenesis, growth, deterioration, and metastasis but is also closely related to the anti-tumor immune response (130). Therefore, regulating the immune response and the expression of inflammatory factors is essential for delaying tumor progression and improving both the survival rate and quality of life for patients.

LJF extract has been found to mitigate TNF-α or IL-6-induced inflammatory responses in hepatocellular carcinoma and macrophage cell lines by inhibiting NF-κB/IL-6/STAT3 signaling, thereby reversing immune suppression (131). NF-κB and STAT3 are crucial transcription factors linking cancer and inflammation (132). The ethanol extract of LJF demonstrates anti-melanoma effects by targeting STAT3 signaling and reshaping the immune microenvironment (20).

Anti-microbiome therapy utilizing LJF bud extract notably reduced polyp burden in ApcMin/+ mice and alleviated intestinal inflammation by shifting macrophages from an M1 to M2 phenotype (133). In cyclophosphamide (CTX)-induced immunosupressed mouse models, LJF polysaccharides significantly enhanced organ index, splenic lymphocyte proliferation, macrophage phagocytosis, and NK cell activity, while restoring serum cytokine levels of IL-2, TNF-α, and IFN-γ, confirming its anti-inflammatory and immunomodulatory properties (134). Moreover, LJF-derived miRNAs have shown anti-tumor immune effects by targeting TGF-β1 to enhance T lymphocyte infiltration in tumor environments (135).

### 5.6 Regulation of autophagy

Autophagy is an evolutionarily conserved, lysosome-mediated biodegradation process that is essential for regulating cell growth and maintaining internal homeostasis (136). Under conditions of cellular stress, autophagy acts prior to apoptosis to preserve cellular equilibrium. Consequently, autophagy is often regarded as a cellular strategy and mechanism for survival in stressful environments (137). It has been reported that autophagic cell death is closely related to many human diseases, especially plays a key role in the initiation and development of cancer (138). In the early stages of tumorigenesis, autophagy functions as a survival pathway and quality control mechanism, inhibiting early tumor development by enhancing antitumor activity. However, once the tumor progresses to advanced stages and encounters environmental stress, autophagy can promote tumor progression by stimulating growth. Therefore, the regulation of autophagy presents a potential intervention strategy for cancer therapy.

In lung cancer studies, luteolin (139) significantly regulated the expression of autophagy-associated proteins, including the accumulation of the microtubule-associated protein light chain-3 (LC3) II, the increase of LC3 puncta, and the enhancement of autophagic flux, which collectively induced autophagy to promote cell death. In contrast, luteolin (140) induced autophagy in human hepatocellular carcinoma Hep3B cells by up-regulating the level of LC3-II protein and down-regulating the level of p62 protein, thereby promoting cancer cell survival.

### 5.7 Activation of related signaling pathways

The occurrence and development of malignant tumors are closely associated with the activation of various signaling pathways. As a traditional Chinese herb, LJF extract and its active components have demonstrated a certain anti-tumor effect in related studies, which is attributed to the regulation of multiple signaling pathways.

In recent years, NF-κB and STAT3 have garnered significant attention in various cancers. Numerous studies have demonstrated that NF-κB is extensively involved in a range of physiological and pathological processes within the body, including inflammatory responses, cell survival, proliferation, differentiation, and tumorigenesis (141, 142). Concurrently, STAT3 plays a crucial role in regulating cell growth and apoptosis by modulating the expression of multiple genes in response to cellular stimuli (132). Research conducted by Ju et al. indicated that (143) luteolin can effectively inhibit NF-κB activity, thereby enhancing the pro-apoptotic effects of JNK on TNF-induced lung cancer cells. Additionally, kaempherol, an active component of LJF, in conjunction with luteolin, was found to inhibit the binding of STAT3 to the claudin-2 promoter region, resulting in decreased expression and proliferation of claudin-2 in A549 cells (144). These findings offer new insights and directions for further exploration in cancer therapy.

The Hippo/YAP pathway is a highly conserved cellular signaling pathway that plays a crucial role in regulating organ size and tumorigenesis (145). In parallel, the Wnt/β-catenin pathway serves as a fundamental molecular mechanism in embryonic development and tissue homeostasis, with its aberrant activation being a significant contributor to the onset and progression of various cancers (146). In our scientific study, we discovered that the mechanism by which LJF exerts its effects against hepatocellular carcinoma (HCC) may be linked to these two pathways (61). Our research data indicated that 3,4-di-O-cafeoylquinic acid, a novel compound isolated from LJF buds, inhibits the proliferation and migration of HCC cells through the suppression of the Hippo/YAP pathway. Additionally, 3,4-di-O-cafeoylquinic acid appears to induce cell cycle arrest in HCC cells by inhibiting the Wnt/β-catenin pathway. These findings suggest that both the Hippo/YAP and Wnt/β-catenin pathways are likely involved in the inhibition of HCC cells induced by 3,4-di-O-cafeoylquinic acid, indicating its potential as a promising therapeutic agent for HCC.

JNK is a significant member of the MAPK family in mammalian cells and has been demonstrated to activate a diverse array of substrates in response to various stimuli, including the regulation of apoptosis, proliferation, tumorigenesis, and inflammation (147). In a screening of the anti-liver cancer active ingredients of LJF extract (protocatechuic acid, chlorogenic acid and luteolin), the researchers found that (62) only protocatechuic acid could activate the JNK and p38 subgroups. Additionally, both the aqueous extract of LJF and protocatechuic acid were found to induce HepG2 liver cancer cell death in a JNK-dependent manner. Another study of hepatocellular carcinoma cells indicated that (148) luteolin, the active ingredient of LJF, enhanced TRAIL-induced apoptosis, potentially mediated by JNK-mediated DR5 expression and autophagy.

Src homology-2 domain-containing protein tyrosine phosphatase-2 (SHP2) is a non-receptor protein tyrosine phosphatase that serves dual roles as both an oncogenic factor and a tumor suppressor in various diseases, making it a promising therapeutic target for cancer treatment. Previous studies have demonstrated that (149) the SHP2/Ras/MAPK signaling pathway is involved in mediating a range of cellular functions, including cancer cell proliferation, apoptosis, and invasion. Notably, total saponins derived from LJF have been reported to inhibit the proliferation, invasion, and apoptosis of thyroid cancer TPC-1 cells by suppressing the activation of the SHP2/Ras/MAPK signaling pathway (87).

## 6 Pharmacokinetics

The determination of the pharmacokinetics of Chinese medicines is of critical importance for optimizing drug delivery and enhancing bioavailability. However, the diversity and complexity of the constituents in Chinese medicines, coupled with the low content of active ingredients in crude extracts and their unique pharmacokinetic properties, present significant challenges for research in this area. Currently, pharmacokinetic studies on LJF remain limited, primarily concentrating on the exploration of active compounds isolated from this plant.

Flavonoids are significant components of LJF. Chen et al. provided data (150) for the investigation of their pharmacokinetics. The flavonoid extracts included rutin, luteolin-7-O-β-D-glucoside (LEG), quercetin-3-O-β-D-glucoside (QEG), and lonicerin. Analysis of the blood plasma concentration-versus-time curve revealed that these components exhibited a similar trend in rats, characterized by rapid absorption and slow elimination. Based on the plasma clearance (CL) data, rutin and LEG demonstrated superior performance compared to QEG. And from the apparent volume of distribution (Vd) data, these components are widely distributed in rats, and their Vd values far exceed the actual blood volume of rats. This method is suitable for the pharmacokinetic study of multi-components of Chinese medicine.

Accumulated studies have demonstrated that chlorogenic acid is not only the principal active ingredient of LJF but also serves as a significant marker for evaluating its quality. Among the investigations into LJF extracts, the pharmacokinetic studies of chlorogenic acid are particularly extensive. Zhou et al. (151) examine meticulously the pharmacokinetics and tissue distribution of chlorogenic acid in rats following oral administration. The results indicated that chlorogenic acid was rapidly absorbed and eliminated in the rats, exhibiting an elimination half-life (T1/2) of approximately 0.8 h. Furthermore, chlorogenic acid was predominantly distributed in the liver, followed by the kidneys, lungs, heart, and spleen in descending order. A separate study by Zhou et al. (152) revealed that the metabolic processes of chlorogenic acid in rats, after intravenous, intramuscular, and gavage administration of LJF extract, conformed to a two-compartmental model. The T1/2 were (0.44 ± 0.08) h, (0.50 ± 0.12) h, and (0.38 ± 0.11) h, respectively. The absolute bioavailability of gavage and intramuscular injection were 37.39% and 94.50%, respectively. These findings provide a scientific basis for further studies on the routes of administration and dosage forms of LJF extract.

Luteoloside serves as a crucial marker for assessing the quality of LJF. Pharmacokinetic studies utilizing scientific bioanalytical methods are significantly important for the further development of luteoloside. Qiu et al. (153) discussed in detail the pharmacokinetics of experimental dogs after intravenous injection of 20mg/kg luteoloside. The results indicated that the T1/2 of luteoloside was approximately 1.21 ± 0.14 h. The mean area under the plasma concentration-time curve from time zero to the last measurable plasma concentration point (AUClast) and the mean area under the plasma concentration-time curve from time zero to infinity (AUCInf) were 785 ± 54.6 and 788 ± 54.1 h ng/mL, respectively. Additionally, CL, mean residence time (MRT), and volume of distribution at steady state (Vss) were 425 ± 30.4 mL/min/kg, 0.62 ± 0.03 h, and 15.8 ± 0.39 L/kg, respectively.

By comparing pharmacokinetic data following a single oral and intravenous dose of isochlorogenic acid C (IAC), Huang et al. (154) found that IAC is rapidly absorbed after oral administration, reaching its maximum concentration (Cmax) at approximately 1 hour. The study also demonstrated that IAC exhibits poor bioavailability in rats, with Cmax and the area under the curve (AUC_0-∞_) positively correlated with dose. Additionally, Zhan et al. analyzed (155) the metabolite composition in rats after oral administration of 4,5-dicaffeoylquinic acid. Their analysis revealed 15 metabolites in plasma and 16 metabolites in urine, encompassing various reaction types such as methylation, hydration, dehydrogenation, reduction, glucuronidation, and sulfate esterification. Furthermore, Luo et al. (156) quantitatively monitored sweroside in the plasma, urine, feces, and bile of rats using the high performance liquid chromatography coupled to ultraviolet detection (HPLC-UV) method. The results indicated that the bioavailability of sweroside was extremely low at 0.31%, which may be attributed to its primary excretion via feces.

Pharmacokinetics focuses on the exploration of the absorption, distribution, metabolism, and excretion processes of drugs within living organisms. Conducting relevant pharmacokinetic studies on LJF extracts and their isolated compounds can elucidate the active ingredients present in LJF and provide valuable reference information for its development and application. However, current studies on the pharmacokinetics of LJF exhibit certain limitations. Most research has been conducted using normal animal models, with relatively few studies addressing pathological conditions. In the future, it is of great clinical significance to study the pharmacokinetics of LJF in pathological models.

## 7 Quality control

LJF is a kind of medicinal and edible plant. The flower is white at first, and then becomes yellow, so it is also called double flower or two flower. This color change represents not only a distinctive biological trait of LJF but also serves as an external indicator of its quality and efficacy, which are closely tied to its economic value. In traditional botanical research (157), the color of LJF is considered a critical criterion for assessing its quality. The initial green-white samples were of the best quality, during which the contents of chlorogenic acid and cynaroside were particularly abundant. Furthermore, the quality of LJF is influenced by various factors, including processing methods, water conditions, pruning techniques, and pesticide residues (158, 159).

According to the Chinese Pharmacopoeia (2015 edition), luteoloside and chlorogenic acid were designated as the quality markers for LJF. Over time, the Chinese Pharmacopoeia (2020 edition) has made more rigorous and detailed provisions on the quality standards of LJF. In addition to retaining the original markers, luteoloside and chlorogenic acid, the pharmacopoeia also incorporated isochlorogenic acid A and isochlorogenic acid C as key indicators for quality control. Furthermore, it clearly states the minimum content standards for various components, including chlorogenic acid, to ensure the stability and controllability of LJF’s efficacy.

Stability and controllable quality are essential prerequisites for the advancement of modern Chinese medicine. The foundation of quality control lies in the thorough analysis of its active ingredients. LJF exhibits a broad spectrum of pharmacological effects due to its intricate chemical composition. Consequently, relying solely on a few iconic components for quality evaluation is insufficient. Drawing on the theory of quality markers (Q-marker) in Chinese medicine, Yuan and his colleagues (160) conducted a comprehensive analysis and prediction of LJF’s quality based on the efficacy, measurability, and specificity of its chemical constituents. They proposed that chlorogenic acid, isochlorogenic acid A, isochlorogenic acid B, isochlorogenic acid C, luteoloside, rutin, sweroside, and secoxyloganin could serve as candidate quality markers for LJF. This study offers a more holistic perspective for the quality assessment of LJF, which holds significant implications for the quality control of Chinese medicines, including LJF.

In recent years, researchers have also worked to establish other quality evaluation methods for LJF. Zhang et al. (161, 162) successfully prepared specific monoclonal antibodies against chlorogenic acid and lignoceroside, named mAb2E2 and mAb3A4, respectively. Based on these monoclonal antibodies, they skillfully developed an indirect competitive enzyme-linked immunosorbent assay (icELISA). This method is not only simple and rapid, but also the results are in high agreement with the HPLC validation results. What’s more, with its excellent performance, icELISA proved to have great potential for detecting chlorogenic acid and lignoceroside content in different LJF herbal samples, providing a new means for quality control of LJF.

In addition, zhang et al. (163) developed an innovative colloidal gold-based lateral flow dipstick immunoassay that enables rapid determination of chlorogenic acid and luteoloside content in LJF, achieving results in just 10 minutes. By visually assessing the color of the test line, one can ascertain whether the sample concentration exceeds the detection limit of the test strip, thereby confirming compliance with quality standards. The convenience and high efficiency of this method provide robust technical support for the rapid detection and quality control of Chinese herbal medicines.

## 8 Toxicity

The current understanding of LJF toxicity is still in its initial stages, with only a limited number of studies conducted on animal models. In an assessment of the acute and subacute toxicity of the ethanol extract of LJF, a single oral dose of 5,000 mg/kg did not result in mortality or any significant visceral pathological changes in rats. Furthermore, continuous administration of a lower dose of 1,000 mg/kg/day over a period of 14 days led to a notable increase in testicular weight in male mice. Additionally, hematological analyses indicated that the extract does not exhibit toxic effects (164).

An in-depth study on the acute toxicity of LJF was conducted by Chi et al. (165) using both intravenous and intraperitoneal administration methods. The experimental results indicated that the median lethal dose (LD50) values ranged from 74.3 g/kg to 84.7 g/kg in mice and rats. Given that the recommended clinical dosage of LJF is 20 g/60 kg, our comprehensive evaluation suggests that its use is relatively safe. Additionally, the acute toxicity of tetraploid LJF was thoroughly assessed by Hu and his colleagues, who (166) compared the LD50 values of aqueous extracts from tetraploid and diploid LJF in mice. The results revealed negligible differences, with LD50 values reaching approximately 412 and 400 times the safe dosage for humans (based on a body mass of 60 kg), respectively. This finding implies that the use of aqueous extracts of tetraploid LJF is relatively safe within a specific dose range. Furthermore, Zhang et al. (167) reported that LJF tablets did not demonstrate significant toxicity in rats at conventional doses following a 30-day feeding experiment.

A study conducted by Huang et al. (168) demonstrated that LJF exhibits an *in vitro* hemolytic effect, particularly when administered as an injection, which poses a potential risk of hemolysis. This effect is attributed to the total saponin content present in LJF. Hemolysis was observed to commence when the mass concentration of total saponins reached 0.6 g/L, with a significant increase in the rate of hemolysis corresponding to higher concentrations. At a mass concentration of 1.2 g/L, the hemolysis rate reached 55.3%. However, in practical applications, Chinese medicine injections typically do not attain such elevated drug concentrations. Consequently, we conclude that it is safe to utilize LJF, either orally or via injection, at conventional dosages.

## 9 Clinical research

### 9.1 Clinical study of LJF extract

Research into the anti-tumor properties of traditional Chinese medicine (TCM) has increasingly focused on LJF as an adjuvant in cancer treatment. Its anti-cancer potential has been validated through both cellular and animal studies, and is progressively being substantiated in clinical settings. Previous clinical trials indicate that the combination of LJF with radiotherapy, chemotherapy and targeted therapies is effective in treating non-small cell lung cancer, liver cancer, nasopharyngeal cancer, and esophageal cancer, etc. This combination therapy not only significantly enhances patient tolerance to radiotherapy and chemotherapy but also effectively mitigates adverse reactions associated with various cancer treatment drugs, including acne-like rashes, hand and foot skin reactions, and oral mucositis.

In the clinical practice of Four Flavor-LJF decoction (169) combined with chemotherapy drugs (Carboplatin and Etoposide) in the treatment of non-small cell lung cancer, the use of LJF not only reduces the frequency of nausea and vomiting, but also inhibits adverse reactions such as neutropenia and thrombocytopenia. It also reduced the incidence of alopecia, neurotoxicity, and muscle and joint pain. Compared with chemotherapy alone, Four Flavor-LJF decoction combined with chemotherapy has better tolerance and safety in the treatment of central NSCLC.

According to the clinical study conducted by Sun et al. (170), the use of LJF in combination therapy for patients undergoing radiotherapy for nasopharyngeal carcinoma significantly reduces the incidence of mild mucosal reactions and effectively alleviates oral and nasal mucosal reactions, thereby improving patient symptoms. In the context of LJF in combination therapy for esophageal cancer radiotherapy (171), the concurrent use of LJF decoction and aluminum magnesium suspension not only significantly enhances treatment efficacy but also shortens the duration of esophagitis. Furthermore, LJF demonstrates immunomodulatory effects in the treatment of radiation-induced esophagitis. In the study by Song et al. (172), the incidence of esophagitis and the levels of inflammatory factors in the treatment group were significantly lower than those in the control group, while the levels of CD3+ T cells, CD4+ T cells, and the CD4+/CD8+ ratio were significantly increased. These findings collectively underscore the positive impact of LJF in the context of cancer radiotherapy.

Most patients treated with epidermal growth factor receptor inhibitors (EGFRIs) experience skin toxic effects, such as acne or papulopustular rash, that seriously affect patients’ quality of life and may lead to treatment interruption. However, a prospective, randomized and controlled study has shown that LJF therapy can effectively reduce acne-like rash caused by EGFRIs. In this study, different treated patients were randomly divided into three groups. The data showed that the incidence of acneiform rash in group A patients treated with LJF prophylacticly was 56.5%, much lower than that in group B (68.1%) and group C (71.7%). Not only that, LJF treatment has a significant effect on reducing the severity of skin toxicity, controlling the time of rash occurrence, reducing the degradation rate and improving the progress (173). This series of research results show that the LJF in the treatment of skin toxicity caused by EGFRIs has significant preventive treatment effect.

As a multi-kinase inhibitor, sorafenib has been shown to prolong the survival of patients and effectively inhibit tumor progression. However, it is often accompanied by a series of adverse reactions during the treatment, one of which is notable is hand-foot skin reaction (HFSR). Patients may have symptoms such as dry skin, rash, itching, hair drying, desquamation, hair loss, and skin induration. These symptoms bring great distress to patients. However, it is encouraging that LJF combined with sorafenib (174, 175) can significantly reduce the incidence of HFSR and improve the tolerance of patients. More importantly, the attenuated efficacy was more significant when LJF and Shengjigao were combined with sorafenib (176).

In the context of clinical research on LJF, we conducted a comprehensive search of authoritative databases. Recognizing the importance of clinical trial registration, we specifically reviewed the Chinese Clinical Trial Registry (https://www.chictr.org.cn/showproj.html?proj=219472) and the International Clinical Trial Register Platform (https://trialsearch.who.int/). Through precise retrieval using the keywords “LJF and cancer,” we identified a notable single-arm clinical study titled “A single-arm clinical study of LJF oral liquid in the treatment of non-small cell lung cancer EGFR-TKIs-associated oral mucositis” with the patent number ChiCTR2400080982. This study examined the potential of combining LJF with EGFR TKIs in the treatment of non-small cell lung cancer, particularly focusing on LJF as an auxiliary agent to mitigate adverse effects. The findings indicate that the use of LJF in treating oral mucositis resulting from EGFR TKIs in non-small cell lung cancer patients may lead to reduced side effects and improved efficacy. Given LJF’s low toxicity, broad availability, and cost-effectiveness, we propose its consideration as a preferable adjunctive therapy in cancer treatment.

### 9.2 Combined anti-tumor efficacy of LJF active components

In recent years, research on Chinese medicine has deepened, leading to widespread recognition of the natural effects of its extracts and active ingredients in the field of anti-tumor therapy. Currently, the combination of conventional therapeutic drugs with natural compounds has emerged as a prominent strategy in cancer treatment. This approach leverages the unique complementary advantages of both modalities, allowing for a reduction in the dosage of chemotherapy drugs while simultaneously minimizing toxicity and side effects. Furthermore, it effectively delays the onset of drug resistance and significantly enhances therapeutic outcomes. Various anticancer active ingredients found in LJF, including chlorogenic acid, luteolin, luteoloside, isochlorogenic acid, rutin, and hyperoside, have been reported to be combined with other chemical therapies and are widely utilized in cancer treatment.

#### 9.2.1 Synergistic effects

In order to improve the effect of cancer treatment, we have successfully combined the active ingredients of LJF with a variety of chemotherapeutic drugs to form an innovative combination therapy. This treatment involves multiple targets and multiple signal transduction pathways, which can effectively enhance the anti-cancer efficacy of chemotherapy drugs.

Oxaliplatin, as a highly effective cytotoxic drug, is often used as the preferred drug for postoperative treatment of colorectal carcinoma. When we combine luteolin with oxaliplatin (177), they can significantly block the cycle progression of cancer cells, and then induce more apoptosis in the periphery of tumor clusters and tumor cell clusters, thus effectively inhibiting tumor growth. In addition, the combination of luteolin and cisplatin (178) not only significantly inhibited cell migration and invasion, but also promoted early apoptosis of cancer cells by down-regulating Bcl-2, and enhanced the anti-proliferative effect of cisplatin on ovarian cancer CAOV3/DDP cells. Further mechanism studies have shown that luteolin can synergistically enhance the anti-tumor effect of 5-fluorouracil (179) on HepG2 and Bel7402 ovarian cancer cells by inducing apoptosis and regulating metabolism. It is worth mentioning that the combination of chlorogenic acid and 5-fluorouracil (180) produced more prominent ROS production and more obvious ERK1/2 inactivation than single treatment, which further mediated the enhancement of 5-fluorouracil-induced inhibition of liver cancer cell proliferation and significantly improved the therapeutic effect of 5-fluorouracil.

In addition to the effective combination with conventional single chemotherapeutic drugs, the anticancer components contained in LJF also show the potential to be combined with new targeted drugs to improve the therapeutic effect. Take lapatinib as an example, which is a tyrosine kinase inhibitor. When lapatinib is used in combination with luteolin (181), they can significantly inhibit the proliferation of breast cancer cells, thereby enhancing the therapeutic effect of lapatinib on human breast cancer. This combination therapy not only increased the sensitivity of SKBR-3, BT-474 and ZR-75-1 cells to treatment, but also up-regulated the gene expression levels of FOXO3 a and NQO1. Therefore, the combination of promising chemotherapeutic drugs with less toxic natural compounds has shown good therapeutic effects and provided new possibilities and directions for cancer treatment.

#### 9.2.2 Sensitization effects

The active ingredient luteolin in LJF can inhibit autophagy and reduce the expression of LC3-II, thereby inhibiting cell viability. At the same time, it can also enhance the sensitivity to cisplatin by inhibiting the expression of RARP1 in epithelial ovarian cancer cells (182).

Studies have shown (183) that the production of cisplatin resistance is closely related to the induced expression of progesterone receptor membrane component (PGRMC1) in ovarian cancer cells. Hyperoside activates AKT signal transduction and Bcl-2 family expression by relying on the autophagy of PGRMC1, induces increased apoptosis, and makes cancer cells more sensitive to cisplatin treatment.

Among the anticancer drugs, Paclitaxel is a commonly used drug for the treatment of breast cancer, and its efficacy is significant. However, high-dose paclitaxel has problems such as relapse resistance and adverse reactions, which has become a major challenge in the treatment of advanced breast cancer. Recently, the study of Sun et al.provided a new possibility for the application of hyperoside in the field of anticancer-as a sensitizer (184). It is speculated that hyperoside may enhance the sensitivity of breast cancer cells to paclitaxel by blocking the pro-inflammatory and pro-survival mechanisms caused by TLR4 activation.

In addition, the active ingredients of LJF can not only enhance the effect of chemotherapy drugs, but also have a significant radiosensitization effect. When rutin is combined with radiation therapy (185), this combination therapy can significantly increase the number of apoptotic colon cancer cells, resulting in more significant cell death. Compared with other treatment groups, the combination therapy also led to changes in mitochondrial membrane potential, increased DNA damage, increased levels of lipid peroxide markers, and decreased antioxidant status. This indicated that rutin played a significant radiosensitizing effect in HT-29 colon cancer cells.

#### 9.2.3 Attenuation effect

Doxorubicin is one of the most effective chemotherapeutic drugs for the treatment of solid tumors. However, its dose-related potential cardiotoxicity may trigger heart failure in patients. Therefore, it is particularly important to develop a drug that has a cardioprotective effect during doxorubicin treatment and enhances its efficacy in cancer cells. Recent studies have shown that (186) when luteolin is combined with doxorubicin, it can significantly inhibit cell proliferation and metastasis, and effectively induce apoptosis. This not only prevents doxorubicin-induced cardiotoxicity, but also enhances its effect against breast cancer.

Oxaliplatin has shown significant efficacy in the treatment of gastric cancer. However, its long-term use can cause side effects such as nephrotoxicity, ototoxicity, neurotoxicity and bone marrow suppression, and may even cause discomfort such as nausea and vomiting. In response to this problem, Li et al. (187) proposed a method for combining rutin with oxaliplatin. This method not only successfully reduced the dose of oxaliplatin, but also significantly reduced its side effects. Compared with oxaliplatin or rutin alone, this combination therapy is more prominent in anti-tumor effect.

Ovarian injury and infertility are common side effects of chemotherapy in female patients with cancer. In the combined treatment of cyclophosphamide and hyperoside (188), the latter shows a unique protective effect. It increases mitochondrial membrane potential by blocking HIF-1α/BNIP3-mediated autophagy activation, thereby increasing follicular reserve and saving fertility in cyclophosphamide-treated mice. This property of hyperoside makes it show great potential in the field of ovarian protection and is expected to help maintain the fertility rate of women receiving chemotherapy.

#### 9.2.4 Anti-drug resistance

Chemotherapy resistance has become a major obstacle to the cure of ovarian cancer. Among them, cisplatin drug resistance is particularly prominent in the treatment of ovarian cancer and has become one of the main challenges. Luteolin, as a natural compound, effectively inhibits the migration and invasion of CAOV3/DDP cells by promoting the process of apoptosis, thereby enhancing the anti-proliferative effect of cisplatin on drug-resistant ovarian cancer cells (178). In addition, luteolin also makes drug-resistant human breast cancer cells sensitive to tamoxifen by inhibiting the expression of cyclin E2 (189).

The long-term drug resistance of tumor cells is an important reason for the recurrence and metastasis of cancer patients. Therefore, it is particularly important to develop alternative therapies against drug resistance. The active ingredient luteolin in LJF may be a new strategy to overcome the resistance of breast cancer patients to tamoxifen. Luteolin has been shown to inhibit the activation of PI3K/AKT/mTOR signaling pathway, effectively induce tumor cell apoptosis, reduce mitochondrial membrane potential, and arrest the cell cycle in the G2/M phase, thereby inhibiting the proliferation of tamoxifen-resistant estrogen receptor -positive breast cancer cells (190).

## 10 Biomedical application

### 10.1 Metal nanoparticles

In recent years, metal nanoparticles (MNPs) have gained significant attention in the biomedical field due to their small size, high area-volume ratio, and excellent reactive activation. Their unique physicochemical properties have made them a focal point of research (191). Currently, it is widely accepted that MNPs synthesized using green plant extracts exhibit superior biocompatibility, medicinal properties, cost-effectiveness, and environmental sustainability, while also demonstrating low toxicity and remarkable anti-cancer effects (192, 193).

LJF, a well-known Chinese herbal plant, is abundant in phenolic acids and flavonoid derivatives, which are associated with effective anti-cancer activities against various cancer cells. Additionally, LJF possesses a strong reducing capability, making it an ideal green material for synthesizing metal nanoparticles. Analysis of existing data indicates that metal nanoparticles prepared with LJF extract as a reducing agent and stabilizer not only offer innovative therapeutic approaches in cancer therapy but also hold significant potential for applications in biomedicine.

Patil et al. (194) found that the majority of gold nanoparticles (AuNPs) synthesized using LJF extract were spherical in shape, with particle sizes ranging from 10 to 40 nm. These nanoparticles exhibited significant cytotoxicity against cervical cancer HeLa cells, effectively inducing apoptosis, which resulted in the death of HeLa cells and inhibited their proliferation. Additionally, Rajivgandhi et al. (195) demonstrated that silver nanoparticles (Ag NPs) synthesized by LJF possess antiproliferative properties against human lung cancer A549 cells. The mechanism of action primarily involves the triggering of ROS production, which subsequently leads to increased apoptotic cell death.

Another study demonstrated that (196) LJF-silver nanoparticles (LJF-Ag NPs), synthesized using LJF as a reducing agent, exhibited a spherical structure similar to that of biological membrane. Supported by comprehensive experimental data, the LJF-Ag NPs significantly enhanced the antioxidant properties, antimicrobial properties, and anticancer activities of LJF extracts. In MTT assays, the synthesized LJF-Ag NPs displayed superior biocompatibility and anticancer effects compared to LJF in cervical cancer HeLa cells, hepatocellular carcinoma HepG2 cells, and breast cancer MDA-MB-231 cells. The nanonization of LJF further amplifies its anticancer activity and presents broader opportunities for clinical trials in the field of anticancer applications. The anti-tumor effect of LJF-metal nanoparticles is worthy of further exploration and research.

### 10.2 Photodynamic therapy

Photodynamic therapy (PDT) has emerged as a cutting-edge technology in cancer treatment, characterized by its minimally invasive nature, portability, high efficiency, low toxicity, and strong targeting capabilities. It is widely utilized in the treatment of various cancers. This therapy employs a specific wavelength of visible light to activate tumor-targeted photosensitizers, which in turn produce ROS that effectively induce apoptosis in tumor cells, thereby achieving therapeutic objectives (197). As the core component of PDT, advancements in the research of photosensitizers are crucial to enhancing the effectiveness of this treatment. While current photosensitizers have demonstrated significant efficacy in clinical practice, they also present certain limitations, including a degree of phototoxicity and restricted availability. Recent studies indicate that natural product photosensitizers offer promising alternatives due to their lower toxicity and potential therapeutic benefits, highlighting their great application potential and future prospects (198).

LJF, recognized as an edible traditional medicine, exhibits notable anti-inflammatory and anti-tumor properties. Prior research has demonstrated that LJF possesses photosensitive characteristics. When exposed to high-pressure xenon lamp irradiation, it generates photodynamic effects that enhance its anti-tumor efficacy. The application of LJF as a natural photosensitizer in photodynamic therapy for Ehrlich′s ascites carcinoma EAC cells results in a significant therapeutic effect (199).

In recent years, several studies have demonstrated that LJF holds significant potential as a photosensitizer in the treatment of lung cancer, effectively enhancing the therapeutic effects of PDT. An incidental study revealed (74) that LJF exhibited notable photocytotoxicity in human lung squamous carcinoma CH27 cells. The underlying molecular mechanism is closely associated with caspase-3-induced apoptosis. As research progressed, Liao et al. (72) confirmed that mitochondrial dysfunction and ERS play crucial roles in the photoactivated LJF-induced apoptosis in CH27 cells, as evidenced by proteomics studies. The involvement of ROS in the apoptosis of CH27 cells induced by photoactivated LJF was established. Additionally, the study indicated that the ethyl acetate fraction of the LJF extract may be a key compound responsible for its photosensitive activity.

LJF is a valuable resource cultivated globally. It is both low cost and safe, demonstrating potential in the treatment of various cancer types. Notably, when combined with PDT, LJF acts as a natural photosensitizer, resulting in a more pronounced therapeutic effect on lung cancer. In conclusion, LJF-mediated PDT is anticipated to emerge as a novel therapeutic approach, offering a new strategy for the prevention and treatment of multiple cancers.

### 10.3 Novel drug delivery systems

The anticancer active ingredients of LJF are facing a series of challenges, including poor permeability, non-targeting and low bioavailability. These defects seriously limit their clinical application. In recent years, with the continuous progress and integration in the field of biomedicine, various advanced nanocarrier systems have emerged. These nanocarriers, including nanoparticles, micelles, liposomes and nanoemulsions, have received extensive attention at home and abroad due to their good biocompatibility, long half-life, permeability, strong targeting and high bioavailability (200–202). In particular, self-microemulsifying drug delivery systems (SMEDDS) and HER2 nanospheres drug delivery systems have made significant progress in improving the delivery and efficacy of LJF active ingredients.

SMEDDS is an attractive carrier system (203), which has excellent performance in improving drug solubility and oral absorption and utilization. For example, Chen et al. (204) successfully delivered chlorogenic acid using a self-microemulsifying drug delivery system. The results showed that chlorogenic acid could be completely released from SMEDDS within 480 minutes. Moreover, the oral bioavailability of chlorogenic acid was significantly increased (249.4% relative to the chlorogenic acid suspension) after oral administration of SMEDDS in mice. More interestingly, SMEDDS significantly changed the tissue distribution of chlorogenic acid, showing better targeting to the kidney, and its relative intake efficiency reached 2.79 (2.79 of the relative intake efficiency). This may be mainly attributed to the fact that SMEDDS increased the absorption of chlorogenic acid and changed its distribution from liver to kidney, thereby slowing down the metabolism of chlorogenic acid and improving its oral bioavailability. Therefore, SMEDDS is considered to be a promising carrier for oral administration of chlorogenic acid.

In addition, biological nanomaterials are gradually being widely studied and applied in the diagnosis and treatment of tumors due to their good physical and chemical properties. Xiao et al. (205) designed a Her-2 antibody-modified nanosphere drug delivery system. This nanospheres can overcome the non-targeted defects of ordinary liposomes, significantly improve the uptake efficiency of luteolin, and play an anti-breast cancer role by significantly inhibiting the proliferation and migration of breast cancer cells and up-regulating the expression of FOXO1. HER2 nanospheres can produce substantial killing effect on tumor cells by enhancing targeting and specificity, which is a very promising drug carrier.

At the same time, chlorogenic acid encapsulated SMEDDS (CHA-SME) can effectively deliver chlorogenic acid to mesenteric lymph nodes for immunotherapy of glioblastoma (206). As an effective targeting carrier, CHA-SME can activate anti-tumor immune response, thereby enhancing the immunotherapy effect of CHA. It may provide new strategies and directions for immunotherapy.

## 11 Homology of medicine and food: prevention and health values

### 11.1 The preventive effect of LJF

In recent years, TCM has garnered significant attention for its distinctive alternative therapies. LJF, as a plant with important medicinal value, not only exhibits anti-tumor potential but also serves multiple adjuvant therapeutic roles in cancer treatment. These roles include preventing postoperative recurrence, alleviating the side effects of radiotherapy and chemotherapy, and enhancing overall treatment efficacy. Moreover, LJF addresses issues such as loss of appetite, insufficient nutritional intake, and decreased immunity associated with tumors. Its unique nutritional properties can provide essential nutrients to help regulate the spleen and stomach, while simultaneously promoting weight gain and improving bodily resistance. Given these benefits, we leverage the LJF medicine and food homology to integrate it into daily porridge or stew, so as to make a variety of therapeutic decoctions (**Table 2**). This approach not only enhances the flavor of porridge or soup but also maximizes the medicinal benefits of LJF, making it both nutritious and effective in preventing and treating diseases, promoting health, and extending life.

**Table 2:** LJF diet prescription.

Name	Ingredient	Methods of Production	Efficacy	Book/Reference
Yinhua Lianzi decoction	*Lonicerae Japonicae Flos* (Jinyinhua) 30g, Nelumbinis Plumula (Lianzi) 50g	Boil the *Lonicerae Japonicae Flos*, remove the slag and boil the *Nelumbinis Plumula*, eat with rock sugar.	Clearing heat and detoxification, strengthening spleen and preventing diarrhea	(207)
Yinhua Pugongying porridge	*Lonicerae Japonicae Flos* (Jinyinhua) 30g, *Taraxaci Herba* (Pugongying) 60g, rice 100g	Add *Lonicerae Japonicae Flos* and *Taraxaci Herba* to water and cook, remove the residue and take the juice, and then cook rice for porridge.	Acute mastitis	*Dietotherapy and health preservation*
Jinyinhua Gancao decoction	*Lonicerae Japonicae Flos* (Jinyinhua) 30g, *Glycyrrhiza* (Gancao)10g, mint 5g	Add 1500 ml water to boil, then reduce the heat to boil for 5 minutes, leave the juice to remove the residue, can be served.	Clearing heat and dissipating fire, suitable for lung heat type constitution	*Waike Shifa*
Shenjin Donggua decoction	Winter melon 400g, Jinhua ham 100g, *Pseudostellariae Radix* (Taizishen) 15g, *Lonicerae Japonicae Flos* (Jinyinhua) 5g, 3 slices of *Ginger* (Shenjiang) and green onion	Add *Pseudostellariae Radix* and *Lonicerae Japonicae Flos* tea bags into the pot, add 1 liter of water and cook for half an hour, then remove the tea bags. Simmer the *Ginger* and ham slices in the soup for about half an hour, then add the winter squash and cook until cooked. Season with a little salt and scallions.	Clearing heat and nourishing	*Shells Guide*
Yinhua Luobo decoction	*Lonicerae Japonicae Flos* (Jinyinhua) 10g, white radish 100g, honey 80g	Peel the radish, wash it, cut it into pieces, mix it with *Lonicerae Japonicae Flos* and honey, steam it in a bowl, take one dose a day, take it three times.	Dissipate wind moisten lung, relieve phlegm and cough	*Family Doctor Newspaper*
Jinyinhua porridge	*Lonicerae Japonicae Flos* (Jinyinhua)15g, rice 100g, white sugar or rock sugar	Wash the *Lonicerae Japonicae Flos*, put it into the pot, add the right amount of water, soak for 5-10 minutes, boil the juice, increase the rice porridge, add white sugar or rock sugar when it is ripe, and then boil it.	Clearing heat and detoxification	*Healthy diet*
Jinyinhua Lvdou porridge	*Lonicerae Japonicae Flos* (Jinyinhua) 30 g, Glycyrrhiza (Gancao) 12g, Vigna Radiata (Lvdou) 100g	Add appropriate amount of water to the pot, add *Lonicerae Japonicae Flos* and *Glycyrrhiza*, boil over high heat, boil over medium and low heat for 3 ~ 5 minutes, add washed mung beans to the soup, boil over high heat again, and boil over medium heat until the mung beans bloom.	Clearing heat and detoxification, eliminating carbuncle and resolving mass, tonifying qi and tonifying middle energizer	*Chinese Herbal Diet*
Danggui Jinyinghua decoction	Black beans 50g, red dates 30g, longan 30g, *Angelicae Sinensis Radix* (Danggui) 5g, *Lonicerae Japonicae Flos* (Jinyinhua) 5g, *Rehmanniae Radix Praeparata* (Shudihuang) 3g, egg 1g, brown sugar 30g	Put black beans, red jujube slices, longan, Chinese medicine package (including *Rehmanniae Radix Praeparata*, *Lonicerae Japonicae Flos*, *Angelicae Sinensis Radix*) into the pot, add 3 times the amount of pure water, and boil over high heat. Clean the eggs, put the shell into the soup, boil for 15 minutes with a small fire, fish out the eggs, peel the eggshell, put them back into the soup and continue to boil for 1.5-2.5 hours with a small fire. When the amount of water in the pot changes from 3 times to 1 times, add the black sugar and stir well until it is completely melted.	Warm uterus	(208)
Jinyinhua Shanzha drink	*Lonicerae Japonicae Flos* (Jinyinhua) 30g, *Crataegus pinnatifida Bunge* (Shanzha) 10g, honey 250ml	Wash the *Lonicerae Japonicae Flos* and *Crataegus pinnatifida Bunge*, put them in the pot, add an appropriate amount of water, and boil them on the fire. Later, the liquid was poured into a small pot, and the liquid was poured out again. Combine the 2 times of liquid medicine, put it into honey, and stir well.	Cold-pungent diaphoresis	(207)
Jinyinhua tea	*Lonicerae Japonicae Flos* (Jinyinhua) 30g, sugar 30g	Wash the *Lonicerae Japonicae Flos*, put it in the pot, and add an appropriate amount of water. Boil the pot on the fire, and then cook for 25 minutes, stop the fire, filter out the slag, add white sugar and stir well.	Clearing heat and detoxification, evacuating wind heat	(209)
Sanhua tea	*Lonicerae Japonicae Flos* (Jinyinhua) 10g, *Chrysanthemi Flos* (Juhua)10g, jasmine 3g, some honey or rock sugar	Add 100-250ml boiling water to cover and soak for 5-10 minutes, it can be used as tea, and honey or rock sugar can be added.	Clearing heat and detoxification	(210)

### 11.2 Modern product development

In recent years, there have been a large number of common food development with LJF as the main component, such as drinks, powder, tea, paste, etc., as shown in **Table 3**.

**Table 3:** The development of ordinary products based on LJF.

Product category	Product name
Paste	Silver fairy toothpaste, LJF ointment, LJF-Menthae Herba mosquito repellent ointment
Powder	LJF ice powder, LJF-Prunellae Spica jelly powder
Tea	LJF tea, LJF-Radix Rhei Et Rhizome tea, LJF-licorice tea, LJF-Menthae Herba tea
Drink	Momordicae Fructus fresh brewing enzyme, LJF cola, LJF dew, LJF soda, Panacis Quinquefolii Radix-Jujubae Fructus-LJF beverage
Candy	LJF candy, LJF ice throat sugar, LJF-Sterculiae Lychnophorae Semen sugar, LJF-Eriobotryae Folium sugar
Meals	LJF fish slippery, LJF vine stewed pork, LJF fried eggs, LJF rolls
Dairy products	LJF Herb Coconut Milk, Guiling Jelly
Liquor	LJF wine

### 11.3 Health food

LJF has a long history of use in healthcare. Historical records, such as the Ben Cao Gang Mu, say that long-term use of LJF can lighten the body and prolong life. A search of the special food information query platform (http://ypzsx.gsxt.gov.cn/specialfood/#/food) of China’s State Administration for Market Regulation reveals that there are currently 80 health products on the market that feature LJF as a primary ingredient, including 75 varieties of “National Food Health Note” and 5 varieties of “National Food Health Word”.Such as Pipa Jinyinhua lozenges (G20120480), Jinyinhua Danshen pearl capsule (G20140309), Fuling Huangqi Jinyinhua capsule (G20190507), Jinyinhua Xiangyuan capsule (G20200152). These LJF-based products are known to not only aid in clearing and nourish throat, eliminating acne, but also enhancing immunity and safeguarding against liver damage.

## 12 Summary and outlook

Cancer is a worldwide disease and all mankind is actively searching for effective alternative treatments. TCM has a rich history of experience in the prevention and treatment of cancer (211–213). LJF, as a commonly used Chinese herbal medicine, has the effects of clearing heat and detoxifying, anti-bacterial and anti-inflammatory. A number of pharmacological studies have shown that LJF extract and its active ingredients (such as chlorogenic acid, luteoloside, rutin, luteolin, hyperoside, isochlorogenic acid) have significant anti-tumor effects. In clinical practice, LJF is often used as an adjuvant drug for cancer treatment. Combined with the results of TCM theory and modern pharmacological research, we speculate that LJF may be a potential alternative method for cancer treatment. Therefore, we have conducted in-depth research on the anti-tumor effect of LJF to highlight its practical application value in clinical practice.

By collating the literature, this article summarizes the active ingredients, anti-cancer mechanisms, pharmacokinetics, quality control, toxicity, clinical research, biomedical applications, and preventive health care functions of LJF. Compared with the previous review, our review more comprehensively expounds the anti-tumor effect of LJF and its clinical application, and provides supplements and updates for related research. Although the research of LJF has achieved rich results, there are still some problems to be solved that we need to explore and overcome.

So far, we have isolated and identified 507 compounds from LJF by plant extraction technology. Including volatile oil, organic acids, flavonoids, iridoids, triterpenoids and triterpenoid saponins. These compounds have shown significant effects in antibacterial, anti-inflammatory, antiviral, antioxidant, hypolipidemic, hypoglycemic, anti-cancer and immune regulation. However, there are still undiscovered trace components in LJF (54). These trace components may have an important impact on the efficacy, safety and clinical application of LJF. Therefore, the isolation and identification of LJF still need further study.

In the process of summarizing the anti-tumor mechanism of LJF, we found that the research on the anti-tumor effect of LJF extract is not enough. Existing anti-tumor evaluations mainly focus on limited active ingredients, and most studies use *in vivo*, *in vitro*, or a combination of both, and the clinical trials involved are very limited. In order to more accurately evaluate the efficacy and reliability of LJF in the field of anti-tumor, we need to carry out more clinical trials to broaden its application potential in clinical treatment.

Through in-depth analysis of pharmacokinetics, we found that LJF extract and its active ingredients showed rapid absorption, slow elimination, wide distribution, and low bioavailability in the human body, which limited its clinical application to a certain extent. Although some nanocarriers have shown positive effects in solving the pharmacodynamics, solubility, targeting and bioavailability of LJF active compounds. However, the number of related studies is limited and there is a lack of sufficient clinical data support. At present, the research on the pharmacokinetics of LJF is mainly carried out in normal animals, and the research on pathological conditions is still insufficient. Considering the complexity of the chemical composition of LJF, its pharmacokinetic process is easily affected by individual differences, drug interactions, disease status and other factors, which makes the research results uncertain. If the pharmacokinetic study of LJF in disease models can be carried out in the future, it will have far-reaching and important clinical significance.

In addition, we must also note that LJF contains a wide variety of chemical components, and its efficacy will be affected by many factors such as origin and processing. Therefore, it is of great significance to construct a set of standardized quality control methods to promote the development of the pharmacodynamics of LJF. The current quality evaluation and control methods are mainly limited to a few compounds such as chlorogenic acid and luteoloside, which cannot fully represent the quality of LJF. Combined with the Q-marker theory of Chinese medicine, we should include more active ingredients into the category of quality markers. In the future, it is suggested that the combination of chemical quality marker evaluation and bioassay should be used to evaluate the quality of LJF more accurately, which can not only provide reference for the quality standard of LJF, but also help us to establish a more comprehensive quality control system, and provide guarantee for the efficacy and safety of clinical medication.

In terms of safety evaluation, there is a lack of sufficient studies regarding the acute toxicity of LJF. However, the 2010 edition of the Chinese Pharmacopoeia recommends a daily dosage of 6 to 15 grams for humans. In practical applications, whether oral or injectable, the drug concentration of LJF usually does not reach such a high level. Therefore, it is safe to use LJF at a conventional dose.

LJF has been widely used in the field of biomedicine due to its strong reducing ability and unique photocytotoxicity. However, the research on the combination of LJF and modern biotechnology is still in its infancy, and its wide application still faces many challenges and problems. Although LJF nanoparticles have many potential application values, their specific utility and safety still need further research and evaluation.

In general, LJF, as a Chinese herbal medicine with rich pharmacological activity, has broad application prospects in cancer treatment. Should future research address the aforementioned related challenges, it will provide more support and guarantee for its clinical application.

## Glossary

OB: Oral Availability
DL: Drug-like properties
KEGG: Kyoto Encyclopedia of Traditional Chinese Medicine
TCM: Traditional Chinese Medicine
CHM: Chinese herbal medicine
IL-1β: Interleukin-1β
IL-6: Interleukin-6
VEGF: Vascular endothlial growth factor
EGF: Epidermal Growth Factor
IL-10: Interleukin-10
TGF-β: Transforming growth factor-β1
Mcl-1: Myeloid cell leukemia-1
JNK: C-Jun N-terminal kinase
ANXA2: Annexin A2
NF-κB: Nuclear transcription factor-κB
PI3K: Phosphatidylinositol 3 kinase
MAPKs: Mitogen-activated protein kinase
KEAP1: Recombinant Kelch Like ECH Associated Protein 1
NRF2: Nuclear Factor erythroid 2-Related Factor 2
ARE: Antioxidant response element
Bcl-2: B-cell lymphoma-2
Bax: Bcl-2-associated X
P53: Tumor Suppressor Protein
EMT: Epithelial-mesenchymal transition
EGFR: Epidermal growth factor receptor
PLCγ: Phospholipase Cγ
ERK1/2: Extracellular signal-regulated kinase 1/2
MMP-9: Matrix metalloproteinase-9
CCR7: Recombinant Chemokine C-C-Motif Receptor 7
MMP-2: Matrix metalloproteinase-2
TNF-α: Tumor Necrosis Factor Alpha
STAT3: Signal transducer and activator of transcription 3
IFN-γ: Interferon-γ
IL-2: Interleukin-2
YAP: , Yes-associated protein
CTGF: Connective tissue growth factor
mTOR: Mechanistic Target Of Rapamycin
AKT: Protein kinase B
NLRP3: NOD-like receptor thermal protein domain associated protein 3
SOX2: SRY-box containing gene 2
XIAP: X-linked inhibitor-of-apoptosis protein
Ras: Rat sarcoma
AgNPs: Silver nanoparticles
PDT: Photodynamic therapy
PCa: Prostate cancer
VRK1: Vaccinia related kinase 1
OPCML: Opioid Binding Protein/Cell Adhesion Molecule Like
CDKs: Cyclin-dependent kinases
CKIs: CDK inhibitors
CDC25C: Cell Division Cycle 25C
Notch-1: Notch (Drosophila) homolog 1
Hes-1: Hes family bHLH transcription factor 1
COX-2: Cyclooxygenase-2
FasL: Fas ligand
CHOP: C/EBP homologous protein
GRP94: Glucose-Regulated Protein 94
GRP78: Glucose-Regulated Protein 78
DR5: Death receptor 5
FOXO3: Forkhead box O3
NQO1: Human NAD (P)H quinone oxidoreductase 1
